# The discovery of potent ribosomal S6 kinase inhibitors by high-throughput screening and structure-guided drug design

**DOI:** 10.18632/oncotarget.1255

**Published:** 2013-08-25

**Authors:** Sylvain Couty, Isaac M. Westwood, Andrew Kalusa, Celine Cano, Jon Travers, Kathy Boxall, Chiau Ling Chow, Sam Burns, Jessica Schmitt, Lisa Pickard, Caterina Barillari, P. Craig McAndrew, Paul A. Clarke, Spiros Linardopoulos, Roger J. Griffin, G. Wynne Aherne, Florence I. Raynaud, Paul Workman, Keith Jones, Rob L.M. van Montfort

**Affiliations:** ^1^ Cancer Research UK Cancer Therapeutics Unit, The Institute of Cancer Research, London, SM2 5NG, UK; ^2^ Division of Structural Biology, The Institute of Cancer Research, London, SW3 6JB, UK; ^3^ Newcastle Cancer Centre, Northern Institute for Cancer Research, School of Chemistry, Bedson Building, Newcastle University, Newcastle upon Tyne, NE1 7RU, UK

**Keywords:** S6 kinase, P70S6K, cancer, inhibitor, structure-based drug design

## Abstract

The ribosomal P70 S6 kinases play a crucial role in PI3K/mTOR regulated signalling pathways and are therefore potential targets for the treatment of a variety of diseases including diabetes and cancer. In this study we describe the identification of three series of chemically distinct S6K1 inhibitors. In addition, we report a novel PKA-S6K1 chimeric protein with five mutations in or near its ATP-binding site, which was used to determine the binding mode of two of the three inhibitor series, and provided a robust system to aid the optimisation of the oxadiazole-substituted benzimidazole inhibitor series. We show that the resulting oxadiazole-substituted aza-benzimidazole is a potent and ligand efficient S6 kinase inhibitor, which blocks the phosphorylation of RPS6 at Ser235/236 in TSC negative HCV29 human bladder cancer cells by inhibiting S6 kinase activity and thus provides a useful tool compound to investigate the function of S6 kinases.

## INTRODUCTION

The 70 KDa ribosomal S6 kinases (S6K) *RPS6KB1* (S6K1) and *RPS6KB2* (S6K2) are key effectors of PI3K/mTOR-regulated signalling, and have been implicated in a variety of human diseases including diabetes and cancer [1-5]. They belong to the family of AGC kinases and are highly homologous with a sequence identity of 83% in their catalytic domains [6]. S6K1, which is the most extensively studied of the two, has been shown to phosphorylate a number of substrates that regulate protein synthesis, including the 40S ribosomal protein S6 (RPS6), and proteins involved in translation, such as the eukaryotic initiation factor 4B (EIF4B) and eukaryotic elongation factor 2 kinase [7].

In turn, S6K1 is activated by phosphorylation of the activation loop residue Thr252 (S6K1 numbering) by PDK1, and by phosphorylation of Thr412, located in the kinase extension region. Phosphorylation of Thr412 is achieved by mTORC1, which is a heterotrimeric complex comprising mTOR, raptor and mLST-8 [1]. However, for full activation of S6K1, these phosphorylation events have to be preceded by phosphorylation of a series of serine and threonine residues in the *C*-terminal autoinhibitory domain and by phosphorylation of Ser394 in the turn-motif [8].

The identification of S6K1 as a potential therapeutic target in oncology is supported by the fact that the S6K1 gene (*RPS6KB1*) is located at human chromosome 17q23, a region that is amplified in 20% of primary breast cancers as measured by comparative genomic hybridisation [9]. In addition, increased expression of S6K1 has been associated with cell transformation and elevated proliferation rates in tumours [10, 11]. Moreover, amplification and overexpression of S6K1 have been linked with poor prognosis and an increased risk of local recurrence [12]. Studies in cultured cells have confirmed that S6K activity is enhanced by mechanisms activating the PI3-kinase/Akt/mTOR pathway, such as loss of the tumour suppressor PTEN, and have shown a positive correlation between S6K activity and tumour growth [13]. In addition, loss of either of the tumour suppressive TSC1/2 complex proteins can result in activation of mTORC1 and S6K. The TSC2 protein acts as a GTPase-activating protein (GAP) towards Rheb (Ras homologue enriched in brain) and promotes conversion of active Rheb-GTP into the inactive form, Rheb-GDP, that can no longer stimulate mTORC1 [14]. Furthermore, S6K1 regulates the turnover of the oncogenic protein MDM2 in the ovarian cancer cell line OVCAR-3 [15]. In DU-145 prostate cancer cells, S6K1 increased survivin expression produced by upstream activation of the pathway, whereas acute ablation of endogenous S6K1 by small interfering RNA down-regulated survivin levels [16]. Finally, increased phosphorylation of S6K and corresponding phosphorylation of RPS6 was observed in HPV16-infected cervical cancer tissue samples [17], and the constitutive activation of S6K1 is associated with cisplatin resistance in human H69 small cell lung cancer cells [18].

Ribosomal S6 kinases are thus interesting therapeutic targets for cancer treatment. In this study we describe a high-throughput screening (HTS) campaign against S6K1 using an amplified luminescent proximity homogeneous assay, and the subsequent hit evaluation for the three hit series we identified. In addition, we report a novel PKA-S6K1 chimeric protein, which was used to elucidate the binding mode of two of the HTS hit series including the improved *C*-6-substituted azabenzimidazole S6K inhibitors. Finally, for the most potent compound from the azabenzimidazole series we demonstrate inhibition of S6K in intact human bladder tumour cells exhibiting loss of expression of the TSC1/2 complex.

## RESULTS AND DISCUSSION

### A PKA-S6K1 Chimera for Structure-Based Drug Design

To date, the available structural data for S6 kinases are limited to three crystal structures of the S6K1 kinase domain. Two structures are of unphosporylated S6K1 and consistent with an inactive state of the kinase. The third is of S6K1 phosphorylated on Thr252 in the activation loop and consistent with a partially activated state of the kinase [19]. The three crystal structures of S6K1 were solved as protein-ligand complexes with the pan-kinase inhibitor staurosporine, but the authors were not able to generate an apo-S6K1 structure, which they attributed to a susceptibility of the apo-enzyme to aggregation upon concentration and upon storage. Whilst the published S6K1 crystal structures provided an exciting first structural insight in S6 kinases, the crystal system might be less applicable to iterative protein-ligand structural studies informing the structure-based design of potent S6K inhibitors.

In order to generate a more robust crystal system suitable for the generation of high-resolution protein-ligand structural data required to facilitate S6K inhibitor design, we decided to develop a S6K1 chimeric protein based on the catalytic subunit of the closely related AGC kinase PKAα (PKA). Chimeric proteins based on PKA have been successfully used in the discovery of PKB inhibitors [20, 21], the analysis of Aurora kinase inhibitors [22], and to study the selectivity determinants of Rho kinase inhibitors [23]. S6K1 and PKA share a sequence identity of approximately 33% in their kinase domains and their ATP-binding sites are nearly identical. Our PKA-S6K1 chimera was created by mutation of five residues in or near the ATP-binding site (F54Y, M120L, V123L, L173M and Q181K) that differ between the two kinases. Analogous to PKA and other PKA-based chimeric proteins, the PKA-S6K1 chimera could be expressed in *E. coli* and the tetra-phosphorylated enzyme purified using a protocol described previously (see Materials and Methods). Co-crystals of purified PKA-S6K1 chimera with PKA inhibitor peptide (PKI, residues 5-24) were successfully grown, routinely diffracted to between 1.5 and 2.0 Å resolution, and ternary complexes with inhibitors could easily be obtained using soaking experiments.

**Figure 1:**
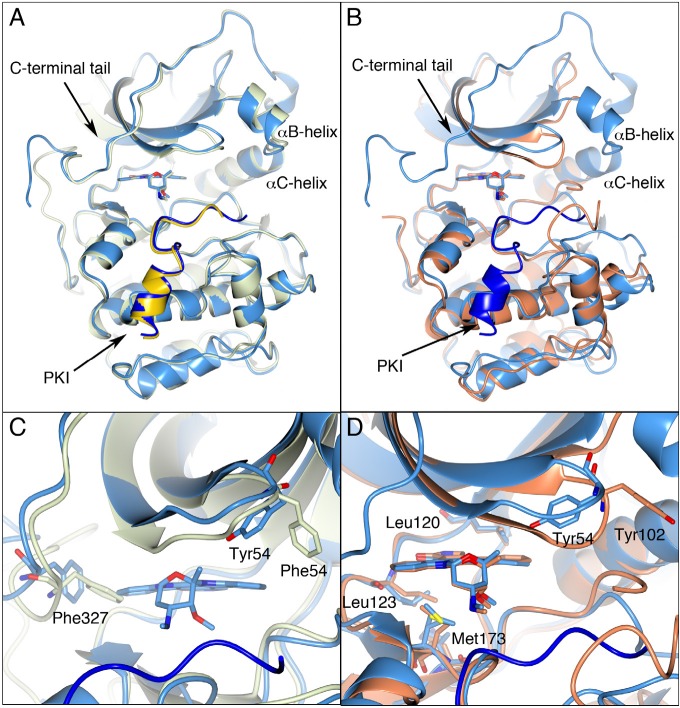
PKA, the PKA-S6K1 chimera and native S6K1 A) Superposition of the staurosporine-bound structures of PKA (PDB code: 1STC) in light green and the PKA-S6K1 chimera in light blue showing the close similarity of the structures. The inhibitor peptide PKI is shown in yellow and blue in the respective structures. The staurosporine molecules are shown in cylinder representation. Relevant secondary structure elements are labelled. B) Superposition of the staurosporine-bound structures of the PKA-S6K1 chimera in light blue and native S6K1 (PDB code: 3A62) in orange showing the similarity in their tertiary structures and the nearly identical binding mode of staurosporine. The inhibitor peptide PKI present in the PKA-S6K1 chimera structure is shown in blue. C) Close-up of the superposition of the staurosporine bound PKA-S6K1 chimera and the adenosine-bound PKA structure (PDB code: 1BKX) which represents an intermediate conformation in the PKA catalytic cycle. The colour scheme is the same as in panel A, but the adenosine molecule bound in 1BKX is not displayed for clarity. Phe327 in the *C*-terminal tail and the aromatic residue at the tip of the P-loop (Tyr54/Phe54) adopt different conformations in the respective structures. D) Close-up of the S6K1 and PKA-S6K1 chimera superposition. The positions of four of the five mutations in the PKA-S6K1 chimera ATP-binding site (Tyr54, Leu120, Leu123, Met173) are highlighted using the PKA-S6K1 sequence numbering. The fifth mutation near the ATP-binding site (Lys181) could not be shown in this orientation. Also shown is Tyr102 in S6K1. All structural figures were made using CCP4MG [48].

To validate the PKA-S6K1 chimera as a structural surrogate for S6K1, we solved the structure of staurosporine bound to the PKA-S6K1 chimera and compared it with the publicly available staurosporine-bound crystal structures of PKA (PDB code: 1STC) and the phosphorylated and partially activated S6K1 (PDB code: 3A62). As expected, the overall conformations of the staurosporine-bound PKA and PKA-S6K1 structures are nearly identical (rmsd 0.51 Å for 330 equivalent *Cα* atoms, Figure 1), except for residues 316 to 320 in the *C*-terminal tail, which adopt a different conformation in the PKA-S6K1 chimera compared to PKA (Figure 1A). In both structures, the PDK1 phosphorylation site in the activation loop (Thr197) is in its phosphorylated state, the activation loop is in an optimal conformation for substrate binding and both structures are ternary complexes with the peptide inhibitor PKI. However, it is important to note that the binding of staurosporine induces substantial conformational changes in the structure of PKA [24] and therefore also in the structure of the PKA-S6K1 chimera. During its catalytic cycle, the conformation of the PKA kinase domain shuttles between an open unliganded conformation, and a closed ATP- and substrate-bound conformation, which together with several intermediate conformations, have been captured in different PKA crystal structures [25]. Both the staurosporine-bound PKA and PKA-S6K1 structures are most similar to an intermediate conformation represented by the adenosine-bound PKA structure (PDB code: 1BKX), in which the P-loop adopts a conformation halfway between the fully open and closed forms of the kinase (Figure 1C). In both staurosporine-bound crystal structures, changes in the side chain conformations of residues lining the respective ATP-binding sites help to accommodate the binding of the bulky staurosporine molecule. The biggest differences include the conformation of the respective Phe54 or Tyr54 residue at the tip of the P-loop, which in both structures is tucked underneath the loop and points towards staurosporine, and the conformation of Phe327 in the *C*-terminal tail, whose side chain is not only shifted, but also rotated by approximately 90 degrees to allow binding of the inhibitor (Figure 1C).

The construct used to elucidate the native S6K1 structure lacks 75 *N*-terminal residues and 126 *C*-terminal residues of the S6K1 sequence. In addition, the S6K1 crystallographic coordinates lack a further 10 *N*-terminal residues and 27 *C*-terminal residues due to disorder of these residues within the crystals used for structure elucidation. Therefore, the native S6K1 crystal structures do not contain the *C*-terminal auto-inhibitory domain and the majority of the characteristic AGC kinase *C*-terminal tail [26], which folds back from the *C*-terminal lobe across the ATP-binding site to the *N*-terminal lobe, is not present. Although the S6K1 construct is considerably shorter than the PKA and PKA-S6K1 chimera sequences, the overall tertiary SK61 structure is very similar to the PKA-S6K1 chimera (rmsd 1.33 Å for 247 equivalent *Cα* atoms, Figure 1B). The most notable differences with the PKA-S6K1 chimera include the disordered *αB*-helix and partially disordered activation loop and *αC*-helix, which are consistent with the low activity of the phospho-Thr252 form of the enzyme. The binding-mode of staurosporine to native S6K1 and the PKA-S6K1 chimera is nearly identical, and the amino acid residues lining the respective ATP-binding sites and contacting staurosporine have very similar side chain conformations. The exception is the conformation of the tyrosine residue (Tyr102 in S6K1 and Tyr54 in PKA-S6K1) at the tip of the P-loop, which extends outward in S6K1, but is folded back underneath the P-loop in the PKA-S6K1 chimera (Figure 1D). This could potentially be a cause of differences in inhibitor-binding between native S6K1 and the PKA-S6K1 chimera, but because of the high similarity of the overall kinase domain structures and the very minor differences in side chain conformations within the respective staurosporine-occupied ATP-binding sites, we concluded that the PKA-S6K1 chimera was a suitable, robust and high-resolution surrogate system to guide our structure-based S6K inhibitor design.

### High-throughput screening of S6K1

**Figure 2:**
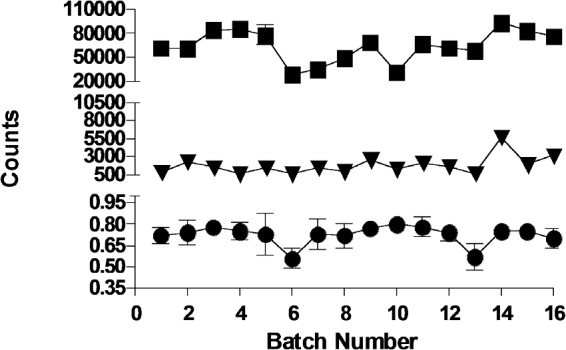
Overall performance of the S6K1 high-throughput screen Alphascreen™ counts (mean ± SD) for total activity wells (■); no enzyme blanks (▼) and Z' factor (●) for 16 batches of 14 plates containing 320 compounds each.

To identify inhibitors of S6K1, an HTS using an AlphaScreen™ kinase assay was carried out which measured the inhibition of the catalytic domain of S6K1 by the reduction in phosphorylation of an RPS6 substrate peptide. A compound library of approximately 67,000 compounds with lead-like properties was screened at a final concentration of 30 μM. With a mean Z' factor of 0.69 ± 0.07 (CV% = 9.3) the overall performance of the HTS was considered to be good (Figure 2). A total of 414 hits were identified yielding an initial hit rate of 0.6%. To exclude compounds active through aggregation, all initial hits were reanalysed using the same assay conditions in the presence of Triton™ X100 [27]. The 67 resulting hits (confirmed hit rate 0.1%) were inspected for chemical tractability, toxicophores and undesired compounds, which left 19 progressible hits of which 12 were commercially available and repurchased for further analysis. The IC_50_ values of 11 of the 12 hits ranged from 0.25 to 22.6 μM, but the remaining compound did not reconfirm in a secondary DELFIA phosphorylation assay and was therefore discarded.

### Hit evaluation

The resulting confirmed HTS hits could be grouped into three chemical series and three singletons. The three chemical series were prioritised for investigation.

**Figure 3:**
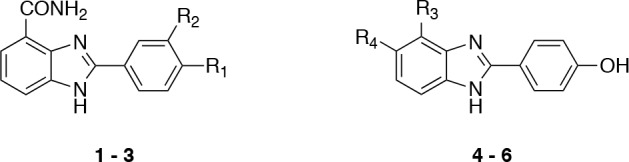
Carboxamidobenzimidazole hits

The first series comprised three carboxamidobenzimidazoles (1 – 3) with respective IC_50_ values of 0.56 μM, 1.04 μM and 10.14 μM (Figure 3 and Table 1). Their favourable physicochemical properties, including molecular weights ≤ 283 Da, ClogP ≤ 2.1, total polar surface areas (tPSA) ≤ 97 Å^2^, and their good ligand efficiencies made them an attractive series for further investigation.

**Table 1:** IC_50_ values and ligand efficiencies of carboxamidobenzimidazole hits 1 – 3

Compound	R_1_	R_2_	R_3_	R_4_	S6K1 IC_50_ (μM)^a^	LE^c^
1	OH	H	-	-	0.56 ± 0.05	0.45
2	H	OH	-	-	1.04 ± 0.16	0.44
3	OH	OMe	-	-	10.1 ± 2.9	0.33
4	-	-	CO_2_H	H	>100^b^	n/a
5	-	-	CONHMe	H	>100^b^	n/a
6	-	-	H	CONH_2_	>100^b^	n/a

a The IC_50_ values were taken from the confirmation DELFIA assays on repurchased samples and are expressed as the mean ± standard error for duplicate measurements.

b Single measurement.

c Ligand efficiencies were calculated using the mean IC_50_ values [49].

**Figure 4:**
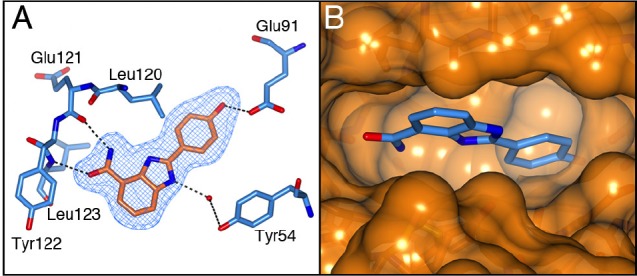
The binding mode of compound 1 A) Crystal structure of the PKA-S6K1 chimera in complex with compound 1. The compound is shown in cylinder representation with orange carbon atoms. PKA-S6K1 amino acids interacting with 1 are displayed in cylinder representation with blue carbon atoms. Solvent atoms are displayed as red spheres and hydrogen bonds are shown as black dashed lines. The electron density shown as a blue mesh is a Fo-Fc omit map contoured at 3σ. B) Superposition of the structure of 1 bound to PKA-S6K1 with the S6K1 structure (PDB code: 3A62) showing that 1 fits in the S6K1 ATP-binding site. Compound 1 is shown in cylinder representation with blue carbon atoms. The S6K1 solvent accessible surface is shown in orange.

The carboxamidobenzimidazoles are known inhibitors of poly(ADP-ribose) polymerase (PARP) with activities in the low nanomolar range [28] and therefore our first aim was to attempt to divorce their S6K1 and PARP activities. It is known that the 4-carboxamide and its intramolecular hydrogen bond to the *N*-3 of the benzimidazole are crucial for PARP activity as this mimics the natural cofactor nicotinamide adenine dinucleotide (NAD^+^). Replacement of the amide by a carboxylic acid group gave compound 4, which is known to be inactive against PARP but also led to loss of activity against S6K1. Similarly, the *N*-methyl amide 5 was inactive against S6K1, as was the regioisomeric 5-carboxamide 6, which is another carboxamidobenzimidazole lacking PARP activity [28]. Initial docking studies and subsequent determination of the crystal structure of compound 1 bound to the PKA-S6K1 chimera showed that the 4-carboxamide group is involved in crucial hydrogen interactions with the hinge region (Figure 4A), which explains the loss of potency upon modifications in this area of the molecule. The phenol group of 1 extends past the medium-sized Leu120 gatekeeper into the selectivity pocket and interacts with the conserved Glu91 in the *αC*-helix. A superposition of the compound 1-bound PKA-S6K1 chimera structure with the S6K1 structure shows that 1 would fit in the ATP-binding site (Figure 4B). However, because the key features of these 2-arylbenzimidazole-4-carboxamides that lead to potent inhibition of S6K1 are also crucial to inhibition of PARP, we did not pursue this series any further.

**Figure 5:**
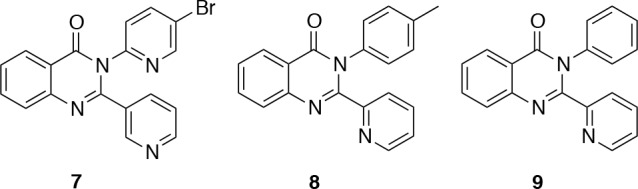
Diarylquinazolinone hits

The second hit series consisted of three 2,3-diaryl(3*H*)-quinazolin-4-ones (Figure 5). Although the compounds in this series had slightly higher ClogP values (2.8–4.0) than the benzimidazoles from series 1, the potencies and ligand efficiencies (Table 2) were still attractive. However, upon resynthesis of these compounds, it became clear that their physicochemical properties are far from ideal. Obtaining reliable assay results was difficult owing to limited aqueous solubility, and despite developing a new synthetic route to this series [29], it proved impossible to develop any directional structure-activity relationships (SAR), which led us to discard this series.

**Table 2:** IC_50_ values and ligand efficiencies of diarylquinazoline hits

Compound	S6K1 IC_50_ (μM)^a^	LE^b^
7	0.26 ± 0.15	0.38
8	1.55 ± 0.92	0.33
9	2.97 ± 1.53	0.33

a The IC_50_ values were taken from the confirmation DELFIA assays on repurchased samples and are expressed as the mean ± standard error for duplicate measurements.

b Ligand efficiencies were calculated using the mean IC_50_ values [49].

**Figure 6:**
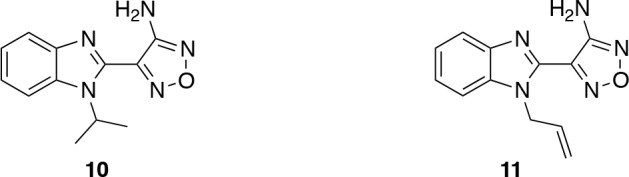
Benzimidazole oxadiazole hits

The third hit series consisted of the two *N*-alkylbenzimidazoles compound 10 and compound 11 which both have an amino-oxadiazole substituent at the *C*-2 position (Figure 6). Their respective IC_50_ values of 340 nM and 7.0 μM translate to good ligand efficiencies (Table 3). At the start of our programme, compounds containing this ring system had been reported as inhibitors of the AGC kinases mitogen and stress-activated protein kinase (MSK1), Rho kinase (ROCK1), and AKT [30-32], and the excellent properties of these hits (ClogP = 2.8, tPSA = 76 Å^2^, Mw = 241/243 Da), led us to select the benzimidazole oxadiazole scaffold as a starting point for our SAR studies. During the course of our work our findings were reinforced by Bandarage *et al*. [33], who also reported benzimidazole oxadiazoles as attractive S6K1 inhibitors.

**Table 3:** IC_50_ values and ligand efficiencies of benzimidazole oxadiazole hits

Compound	S6K1 IC_50_ (μM)^a^	LE^b^
10	0.34 ± 0.14	0.50
11	6.98 ± 2.07	0.40

a The IC_50_ values were taken from the confirmation DELFIA assays on repurchased samples and are expressed as the mean ± standard error for duplicate measurements.

b Ligand efficiencies were calculated using the mean IC_50_ values.

### Exploration of the benzimidazole *N*-1 position

**Scheme 1:**
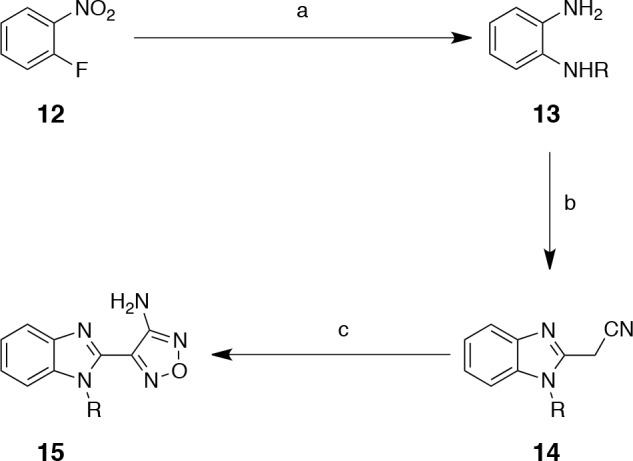
Preparation of 2-aminooxadiazole benzimidazole derivatives Reagents and conditions: (a) i. RNH_2_, KF, K_2_CO_3_, 50-100 °C, ii. Pd(10%)/C, EtOH, rt or SnCl_2_·2H_2_O, reflux (77-86%, 2 steps); (b) ethyl cyanoacetate, 195 °C, 2 h (43-70%); (c) NaNO_2_, 6 M aq. HCl, MeOH then 6 M aq. NaOH, 50% aq. NH_2_OH, reflux (10-45%).

It was initially decided to investigate simple alkyl modifications at the *N*-1 position of the benzimidazole scaffold. Several analogues were prepared according to well-established literature methods (Scheme 1) [33].

**Figure 7:**
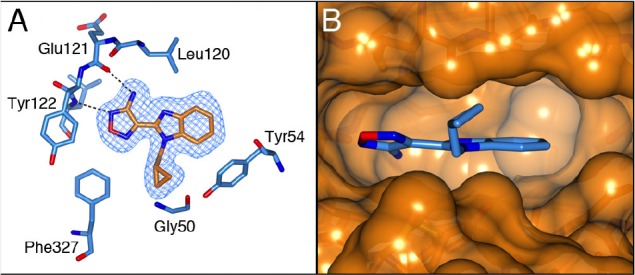
Binding mode of compound 15e A) Crystal structure of the PKA-S6K1 chimera in complex with compound 15e. The compound is shown in cylinder representation with orange carbon atoms. PKA-S6K1 amino acids interacting with 15e are displayed in cylinder representation with blue carbon atoms. Hydrogen bonds are shown as black dashed lines. The electron density shown as a blue mesh is a Fo-Fc omit map contoured at 3σ. B) Superposition of the structure of 15e bound to PKA-S6K1 with the S6K1 structure (PDB code: 3A62) showing that 15e fits well in the S6K1 ATP-binding site. However, due to the flexibility in the S6K1 P-loop and disorder in the *C*-terminal tail it cannot be confirmed if the hydrophobic pocket binding the cyclopropyl group of 15e in the PKA-S6K1 chimera is present in native S6K1. Compound 15e is shown in cylinder representation with blue carbon atoms. The S6K1 solvent accessible surface is shown in orange.

In agreement with the results of Bandarage *et al.*, compounds 15b, 15c and 15e with the respective ethyl, cyclopropyl and cyclopropylmethyl substituents were most potent, but the *N*-methyl analogue compound 15a was about six-fold less active (Table 4). In addition, incorporation of the larger benzyl substituent (15d) resulted in complete loss of biochemical activity. The crystal structure of the PKA-S6K1 chimera bound to 15e (Figure 7a) shows that its cyclopropylmethyl group fits snugly in a hydrophobic pocket formed by Gly50, Tyr54, Val57, and Phe327, as proposed by Banderage *et al*. [33], and suggests that this pocket is too small to accommodate the benzyl group of 15d. Superposition of 15e on the staurosporine-bound S6K1 structure shows that the compound also fits in the native S6K1 ATP binding site, but owing to the disorder in the S6K1 *C*-terminal tail and the different conformation of the P-loop, the presence of the hydrophobic pocket binding the cyclopropyl group could not be verified (Figure 7b).

**Table 4:** IC_50_ values and ligand efficiencies for *N*-1 substituted compounds 15 (Scheme 1)

Compound	R	S6K1 IC_50_ (nM)^a^	LE^e^
15a	Methyl	194^d^	0.58
15b	Ethyl	59.8 ± 32.4^b^	0.59
15c	Cyclopropyl	36.6 ± 7.0^b^	0.57
15d	Benzyl	>100000^c^	n/a
15e	Cyclopropyl methyl	19.8^d^	0.56

a The IC_50_ values were determined with a mobility shift assay.

b Mean ± standard deviation from triplicate measurements.

c Duplicate measurement.

d Single measurement.

e Ligand efficiencies were calculated using the mean IC_50_ values.

**Scheme 2:**
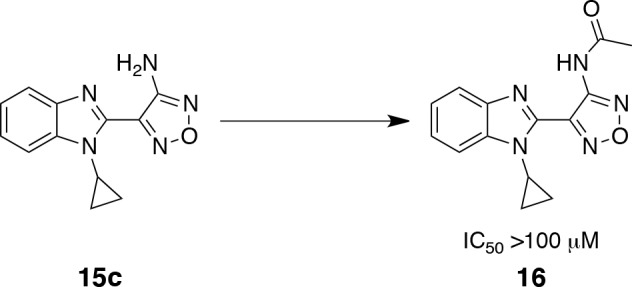
Preparation of 2-acetamide-oxadiazole derivative 16 Reagents and conditions: AcCl, rt (70%).

Acetylation on the exocyclic amino group of the aminooxadiazole moiety renders this series inactive against S6K, as shown by compound 16, with an IC_50_ value of greater than 100 μM (Scheme 2). This is consistent with the binding mode observed in the 15e-bound PKA-S6K1 structure, in which the oxadiazole group binds to the hinge by the formation of a hydrogen bond between its *N*-2 atom and the backbone amide group of Leu123 and through a hydrogen bond between the exocyclic amino group with the backbone carbonyl of Glu121. Although the closely related acetylated compound 16 could in principle still form these hydrogen bonds, in this binding mode the acetyl group would clash with the gatekeeper residue Leu120 and Val104, situated at the bottom of the ATP-binding site.

### Exploration of the benzimidazole *C*-5 and *C*-7 positions

**Scheme 3:**
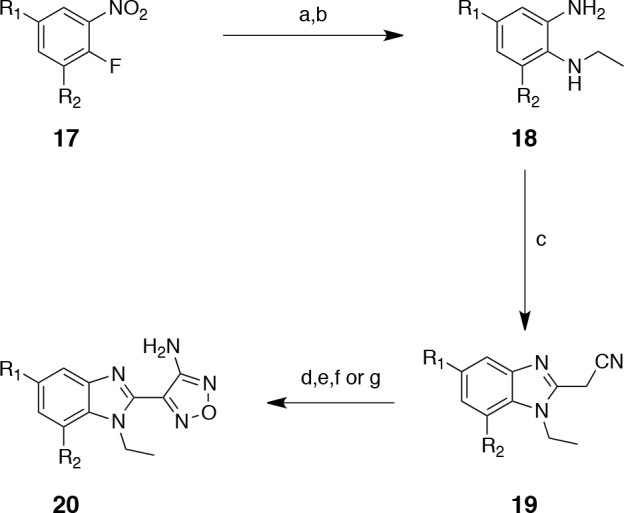
Preparation of *C*-5 and *C*-7 substituted benzimidazoles; Reagents and conditions: (a) EtNH_2_, THF (quant.); (b) Na_2_S_2_O_4_, EtOH, reflux (90%); (c) i. CDI, cyanoacetic acid, THF, reflux; ii. acetic acid (81%, 2 steps); (d) NaNO_2_, 6 M aq. HCl, MeOH then 6 M aq. NaOH, 50% aq. NH_2_OH, reflux (54%); (e) i. *n*-Buli, B(OMe)_3_, THF then aq. H_2_O_2_ (41%); ii. MeI, K_2_CO_3_, acetone (80%); (f) i. trifluoroacetamide, CuI, *N*,*N'*-dimethylethylenediamine, K_2_CO_3_, dioxane (25%); ii. MeI, KOH, acetone, reflux then 5% aq. NaOH, EtOH, reflux (70%); (g) PhB(OH)_2_ or cyclopropylboronate ester or 3-pyridine boronic acid, Pd(PPh_3_)_4_, 1 M aq. NaOH, DME, 150 °C (71%,15% and 25% respectively).

Modifications on the benzene ring at the *C*-5 and *C*-7 positions were investigated as shown in Scheme 3. Preparation of the requisite bromo-precursors 20a and 20f (Scheme 3) allowed the synthesis of analogues incorporating phenyl (20b), cyclopropyl (20c), methoxy (20d) and methylamino (20e) on the R_1_ position and 3-pyridyl on the R_2_ position (20f, Scheme 3). However, unfortunately none of these modifications yielded an improvement in potency (Table 5).

**Table 5:** IC_50_ values and ligand efficiencies of *C*-5 and *C*-7 substituted benzimidazoles (Scheme 3)

Compound	R_1_	R_2_	S6K1 IC_50_ (nM) ^a^	LE^c^
20a	Br	H	66.4 ± 5.6	0.55
20b	Ph	H	1640 ± 530	0.35
20c	cyclopropyl	H	334 ± 25.1	0.45
20d	OMe	H	63.4^b^	0.52
20e	NHMe	H	148^b^	0.50
20f	H	Br	303 ± 21	0.50
20g	H	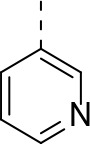	352 ± 173	0.39

a The IC_50_ values were determined with a mobility shift assay and expressed as the mean ± standard deviation for triplicate measurements.

b Single measurement.

c Ligand efficiencies were calculated using the mean IC_50_ values.

### Azabenzimidazoles

**Scheme 4:**
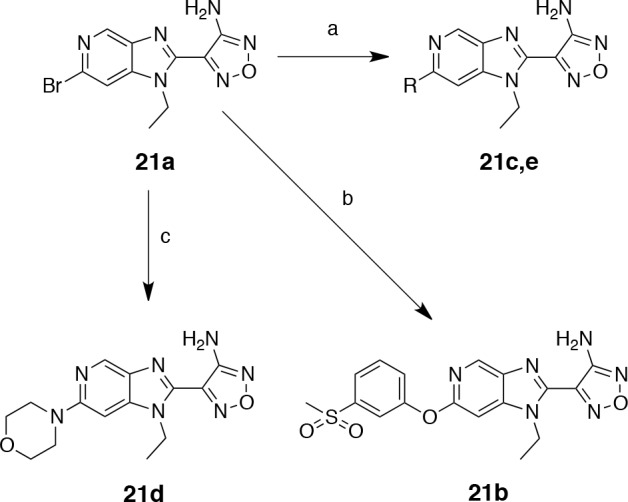
Preparation of azabenzimidazole derivatives Reagents and conditions: (a) RB(OH)_2_, Pd(PPh_3_)_4_, 1 M aq. NaOH, DME, 150 °C microwave [R = Me (25%), R = Ph (59%)]; (b) methyl-(3-hydroxyphenyl)sulfone, Cs_2_CO_3_, Cu (10 mol%), Fe(acac)_3_ (30 mol%), DMF, 150 °C, microwave (17%); (c) morpholine, NMP, 160 °C microwave (51%).

In order to diversify this scaffold, we chose to replace the benzimidazole ring with an azabenzimidazole. Azabenzimidazole aminooxadiazoles have been reported as potent Rho kinase inhibitors and upon analysis of their selectivity profile were also found to have a strong affinity for S6K [34]. Therefore we synthesised a limited set of azabenzimidazole analogues from the bromo-precursor 21a using the synthetic route reported (see Scheme 4) [34]. Interestingly, 21a (Table 6) was equipotent with the *N*-1 cyclopropyl-substituted benzimidazole oxadiazole 15c. Introduction of a phenoxy group in the *C*-6 position (21b) conferred some activity as already suggested by the selectivity studies on Rho kinase. Substitution with a phenyl group at *C*-6 (21c) turned out to be detrimental and abolished all affinity for S6K1. An attempt to improve the physicochemical properties by introducing amino-side chains such as a morpholine group *α*-to the *N*-atom (21d) also proved unsuccessful. However, the methyl-substituted compound 21e represented the most potent inhibitor within this series, and with its ligand efficiency of 0.6 could prove to be an attractive tool compound for further S6K studies (Table 6).

**Table 6:** IC_50_ values and ligand efficiencies of azabenzimidazoles (Scheme 4)

Compound	R	S6K1 IC_50_ (nM) ^a^	LE^d^
21a	Br	19.7 ± 2.6	0.59
21b	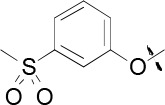	103 ± 23^b^	0.34
21c	Ph	>100000	n/a
21d	morpholine	110^c^	0.42
21e	Me	17.4 ± 6.0	0.60

a The IC_50_ values were determined with a mobility shift assay and expressed as the mean ± standard deviation for triplicate measurements.

b Mean value from duplicate measurement ± standard error.

c Single measurement.

d Ligand efficiencies were calculated using the mean IC_50_ values.

**Figure 8:**
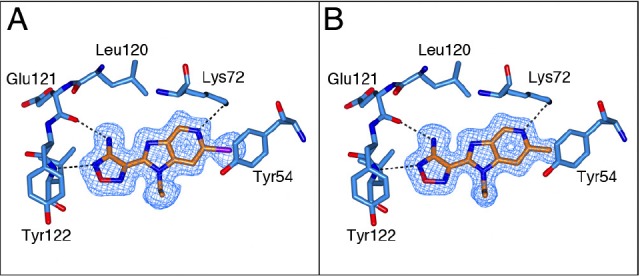
The binding mode of azabenzimidazoles 21a and 21e A) Crystal structure of the PKA-S6K1 chimera in complex with the synthetic precursor compound 21a. B) Crystal structure of the PKA-S6K1 chimera in complex with compound 21e. The compounds are shown in cylinder representation with orange carbon atoms. PKA-S6K1 amino acids interacting with the respective compounds are displayed in cylinder representation with blue carbon atoms. Hydrogen bonds are shown as black dashed lines. The electron density shown as a blue mesh are Fo-Fc omit maps contoured at 3σ.

The binding mode of both the synthetic precursor 21a and 21e was determined by solving the crystal structures of these inhibitors in complex with the PKA-S6K1 chimera (Figure 8). Both compounds bind to PKA-S6K1 in the same orientation as the benzimidazole oxadiazoles with the azabenzimidazole nitrogen forming an additional hydrogen bond interaction with the catalytic Lys72, which in turn forms the conserved salt bridge with Glu91 located in the *αC*-helix. In both structures the respective bromo and methyl *C*-6 substituents pack against the side chain ring of Tyr54, located at the tip of the P-loop and are found to be involved in protein-inhibitor interactions in other kinases such as PKB [21].

### Comparison of the binding modes of identified S6K1 inhibitors

The PKA-S6K1 inhibitor structures described in this paper are most similar to the intermediate adenosine-bound PKA conformation (PDB code: 1BKX). In all structures, Phe327 adopts a conformation similar to the one observed in PKA and consistent with shielding the bound ligand from the solvent.

**Figure 9:**
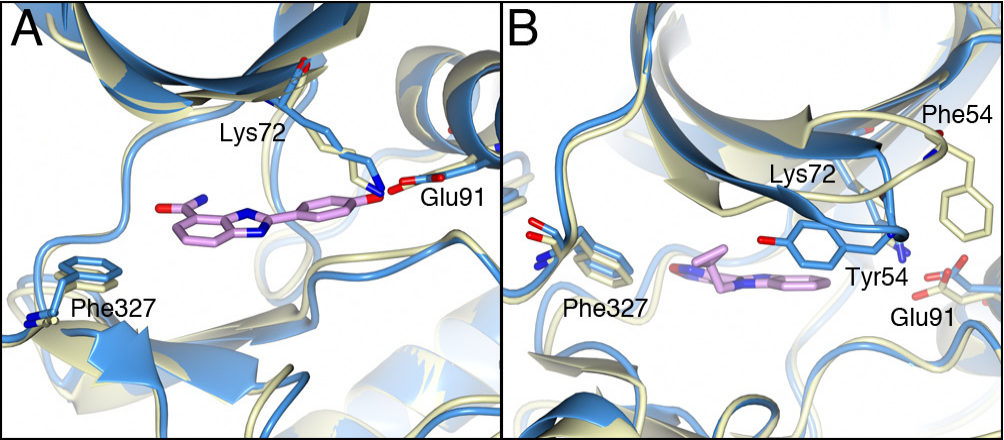
Comparision of 4-carboxamidobenzimidazole and benzimidazole-oxadiazole with PKA A). Superposition of the compound 1 bound PKAS6K1 structure (light-blue) with the intermediate adenosine-bound PKA structure (PDB code: 1BKX, lemon yellow). Compound 1 is shown in pink. The phenol group of compound 1 extends into the selectivity pocket. B) Superposition of the compound 15e bound PKA-S6K1 structure (light blue) with 1BKX (lemon yellow). Compound 15e is shown in pink. Tyr54 at the tip of the P-loop of the PKA-S6K1 chimera swings towards the benzimidazole core scaffold of the inhibitor. In both inhibitor-bound structures Phe327 is in a similar conformation as observed in the adenosine-bound structure.

The carboxamidobenzimidazole 1 is the only inhibitor which binds in the selectivity pocket, whereas the benzimidazole-oxadiazole 15e and azabenzimidazole-oxadiazoles 21a and 21e bind in a very similar manner exploiting the small hydrophobic pocket formed by Phe327 from the C-terminal tail, Gly50 and Tyr54 from the P-loop (Figure 9A). Interestingly, in the benzimidazole and azabenzimidazole inhibitors the aromatic Tyr54 at the tip of the P-loop closes in over the inhibitor and packs against the hydrophobic side of the inhibitors. In compounds 21a and 21e, Tyr 54 interacts with the respective bromine and methyl groups, but the effect is most pronounced for compound 15e, which is unsubstituted in this position allowing Tyr54 to interact directly with the core scaffold of the inhibitor (Figure 9B). The closing of the P-loop over these inhibitors effectively shields them from the solvent, which together with the hydrophobic stacking interactions is likely to contribute to the potency of these inhibitors.

### Cellular activity of azabenzimidazole 21e (CCT239066)

**Figure 10:**
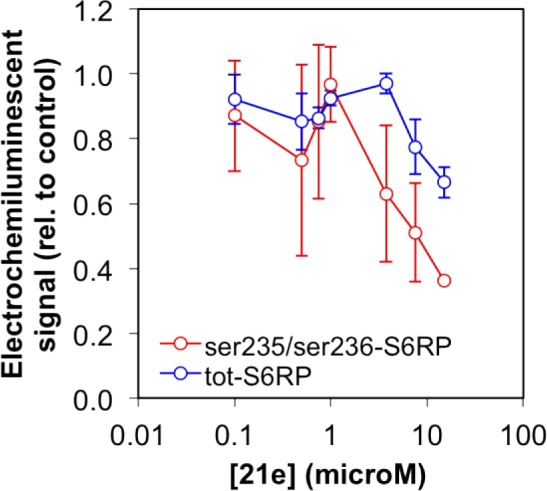
Inhibition of RPS6 phosphorylation Results from an electrochemiluminscent ELISA assay showing the dose-dependent inhibition of phosphorylation of RPS6 on serine 235 and serine 236 in HCV29 human bladder cancer cells following a two hour exposure to compound 21e.

We examined the activity of the substituted-azabenzimidazole 21e (CCT239066) in a human bladder carcinoma line (HCV29) that has activated mTOR signalling as a result of losing expression of the TSC complex which allows activation of mTORC1 [35]. CCT239066 (21e) inhibits S6K1 with IC_50_ values of 17.4 ± 6.0 nM and S6K2 at 300 ± 102 nM respectively and demonstrated 50% tumour growth inhibition (GI_50_) at a concentration of 3.5 μM following 96 hours continuous exposure. CCT239066 (21e) also inhibited phosphorylation of a biomarker of S6K1 activity, phosphorylation of RPS6 at Ser235/236 (Figure 10) in intact cells. The EC_50_ for 50% inhibition of Ser235/236 phosphorylation following 2 hours exposure was 9.1 ± 1.5 μM (n=3) compared to IC_50_ values > 15 μM for AKT-Thr308, AKT-Ser473, GSK3b-Ser9 and S6K-Ser421/424 as determined by electrochemoluminescent ELISA in intact cells.

In conclusion, we have identified three different chemical series of S6 kinase inhibitors. Optimisation of the benzimidazole oxadiazole series resulted in a potent, ligand efficient azabenzimidazole S6K1/S6K2 inhibitor with good physicochemical properties and cellular activity. In addition, we developed a novel robust crystallography system based on a PKA-S6K1 chimera and experimentally determined the binding mode of two of the three inhibitor series, including the improved and cellularly active azabenzimidazole inhibitor. The availability of both a robust crystallography system and cellularly active tool compounds will aid the future design of potent and selective S6K inhibitors.

## METHODS

### High-throughput screening of S6K1

The catalytic domain of S6K1 was screened against our in-house HTS library of lead-like compounds using an AlphaScreen™ kinase assay in which inhibition of S6K1 was measured by a reduction in the phosphorylation of a S6 peptide. Compounds were screened at a final concentration of 30 μM and 0.3% *(v/v)* DMSO by dispensing 3 μL compound solution from a source plate containing the compounds at a concentration of 200 μM in 2% *(v/v)* DMSO, into the central 320 wells of a polypropylene 384-well assay plate (#781287; Greiner Bio-One, Frickenhausen, Germany). 0.3% (*v/v*) DMSO was used as a vehicle control. 17 μL of the reaction mixture containing 1 nM S6K1 (#14-486; Millipore, Billerica, MA, USA), 30 μM ATP and 250 nM S6 peptide (Biotin-KRRRLSSLRASTSKSESSQKI, synthesised by J. Metcalfe, ICR) in assay buffer consisting of 40 mM HEPES pH 7.5, 10 mM MgCl_2_, 1 mM DTT and 0.02% *(v/v)* Tween 20, was added to the compound plate and incubated for an hour at room temperature. Each 384 well plate contained 32 control wells for total enzyme activity, no enzyme blanks and the positive control, staurosporine at a final concentration of 20 nM. The reaction was stopped by the addition of 80 μL of 50 mM EDTA pH 7.5. An aliquot containing 10 μL of the assay solution was transferred to a white OptiPlate™ (#6007299; PerkinElmer Life Sciences, Waltham, MA, USA). AlphaScreen™ streptavidin-coated donor beads, protein A-conjugated acceptor beads (#6760617M; PerkinElmer Life Sciences) and anti-phospho S6 antibody (#2211; Cell Signalling Technology Inc., Danvers, MA, USA) were diluted in bead buffer comprising 40 mM HEPES pH 7.5, 40 mM NaCl, 5% *(v/v)* glycerol and 0.125% BSA and added to the OptiPlate™ resulting in a final concentration of 0.1 nM antibody and 10 μg/mL beads. Plates were sealed and incubated overnight at room temperature in the dark before being read on the Fusion™ Multilabel Reader (PerkinElmer Life Sciences). Using a 300 ms excitation at 680 nm and 700 ms per well reading time at 520-620 nm the signal was expressed in counts per second. A MiniTrak™ V (PerkinElmer Life Sciences) was used for compound and reagent addition. Primary screening data were analysed in ActivityBase (IDBS, Guildford, UK). Compounds with a percentage inhibition of 50% or better were classed as initial hits. All hits were cherry-picked and re-assayed in triplicate to confirm activity. In addition, the hits were tested for aggregation by assaying them in triplicate with 0.01% *(v/v)* Triton™ X-100 added to the assay buffer.

### General synthetic chemistry

Reactions were carried out under nitrogen or argon when necessary. Organic solutions were dried over MgSO_4_. Starting materials and solvents were purchased from commercial suppliers and were used without further purification. Microwave reactions were carried out using a Biotage Initiator 60 microwave reactor. Flash silica chromatography was performed using Merck silica gel 60 (0.025-0.04 mm) and ethylacetate/petroleum spirit (40-60). Ion exchange chromatography was performed using Isolute Flash SCX-II (acidic). ^1^H NMR spectra were recorded on a Bruker AMX500 instrument using internal deuterium lock. ^13^C NMR spectra were recorded at 125 MHz on a Bruker AMX500 instrument. Chemical shifts (δ) are reported relative to TMS (δ=0) and/or referenced to the solvent in which they were measured. Combined HPLC-MS analyses were recorded using a Waters Alliance 2795 separations module and Waters/Micromass LCT mass detector with electro spray ionization (+ve or -ve ion mode as indicated) and with HPLC performed using Supelco DISCOVERY C18, 50 mm × 4.6 mm or 30 mm × 4.6 mm i.d. columns, at a temperature of 22 °C with gradient elution of 10-90% MeOH/0.1% aqueous formic acid at a flow rate of 1 mL/min and a run time of 6 min. Compounds were detected at 254 nm using a Waters 2487 dual λ absorbance detector. All tested compounds gave 95% purity as determined by this method. All purified synthetic intermediates gave 95% purity as determined by this method except where indicated in the text. High-resolution mass spectra were measured on an Agilent 6210 ToF HPLC-MS with a Phenomenex Gemini 3 μm C18 (3 cm × 4.6 mm i.d.) column.

The synthesis of the key oxadiazole-substituted benzimidazoles and azabenzimidazoles 15a-e, 16, and 21b-e from their respective precursors is described below. For details of the synthesis of all precursors and compounds 20a-g see the supplementary information.

### 4-(1-Methyl-1*H*-benzoimidazol-2-yl)-1,2,5-oxadiazole-3-ylamine (15a)

Benzimidazole 14a (342 mg, 2.00 mmol) was diluted in MeOH (2 mL) and aqueous HCl (6 M, 1.5 mL). Solid NaNO_2_ (166 mg, 2.40 mmol, 1.2 equiv.) was added portionwise over 10 min. After the addition, the reaction mixture was made basic with the addition of aqueous NaOH (6 M, 1.5 mL) and hydroxylamine (50% aqueous solution, 0.68 mL, 10.27 mmol, 5.1 equiv.) was added. The reaction mixture was heated to reflux for 4 h, cooled and filtered (H_2_O and MeOH). Purification of the resulting solid by chromatography (SiO_2_, petroleum spirit/EtOAc: 70/30) afforded the title compound as a light yellow solid (254 mg, 45%). ^1^H NMR (500 MHz, DMSO-*d*_6_): *δ*=7.82 (d, *J*=8.1 Hz, 1H), 7.76 (d, *J*=8.1 Hz, 1H), 7.44 (ddd, *J*=1.0, 7.1, 8.1 Hz, 1H), 7.35 (ddd, *J*=1.0, 7.1, 8.1 Hz, 1H), 6.99 (br s, 2H), 4.16 ppm (s, 3H); ^13^C NMR (125 MHz, DMSO-*d*_6_): *δ*=156.1 (s), 141.5 (s), 140.9 (s), 138.4 (s), 135.8 (s), 124.4 (d), 123.0 (d), 119.8 (d), 111.1 (d), 32.2 ppm (q); LC-MS: *m/z*: 216 [*M*+H]^+^, Rt = 4.39 min; HR LC-MS: *m/z* calcd for C_10_H_10_N_5_O: 216.0879 [*M*+H]^+^; found: 216.0878.

### 4-(1-Ethyl-1*H*-benzoimidazol-2-yl)-1,2,5-oxadiazole-3-ylamine (15b)

The benzimidazole 14b (574 mg, 3.10 mmol) was dissolved in MeOH (2.5 mL) and aqueous HCl (6 M, 2.3 mL). Solid NaNO_2_ (256 mg, 3.72 mmol, 1.2 equiv.) was added portionwise over 10 min. After the addition, the reaction mixture was made basic with aqueous NaOH (6 M, 2.3 mL) and hydroxylamine hydrochloride (226 mg, 3.25 mmol, 1.05 equiv.) was added. The reaction mixture was heated to reflux for 2 h, cooled and filtered (H_2_O and MeOH). Purification of the resulting solid by chromatography (SiO_2_, petroleum spirit/EtOAc: 70/30) afforded the title compound as a yellow solid (62 mg, 9%). ^1^H NMR (500 MHz, DMSO-*d*_6_): *δ*=7.82 (d, *J*=7.8 Hz, 1H), 7.79 (d, *J*=7.8 Hz, 1H), 7.43 (m, 1H), 7.36 (m, 1H), 6.97 (br s, 2H), 4.70 (m, 2H), 1.39 ppm (t, *J*=6.3 Hz, 3H); ^13^C NMR (125 MHz, DMSO-*d*_6_): *δ*=156.1 (s), 141.7 (s), 140.1 (s), 138.1 (s), 134.7 (s), 124.5 (d), 123.1 (d), 120.0 (d), 111.0 (d), 40.1 (t), 14.8 ppm (q); LC-MS: *m/z*: 230 [*M*+H]^+^, Rt = 4.65 min; HR LC-MS: *m/z* calcd for C_11_H_12_N_5_O: 230.1036 [*M*+H]^+^; found: 230.1036.

### 4-(1-Cyclopropyl-1*H*-benzoimidazol-2-yl)-1,2,5-oxadiazole-3-ylamine (15c)

The benzimidazole 14c (318 mg, 1.61 mmol) was diluted in MeOH (1 mL) and aqueous HCl (6 M, 1.5 mL). Solid NaNO_2_ (135 mg, 1.93 mmol, 1.2 equiv.) was added portionwise over 10 min. After the addition, the reaction mixture was made basic with aqueous NaOH (6 M, 1.5 mL) and hydroxylamine (50% aqueous solution, 0.55 mL, 8.30 mmol, 5.1 equiv.) was added. The reaction mixture was heated to reflux for 3 h, cooled and filtered (H_2_O and MeOH). Purification of the resulting solid by chromatography (SiO_2_, petroleum spirit/EtOAc: 70/30) afforded the title compound as a green solid (154 mg, 40%). ^1^H NMR (500 MHz, DMSO-*d*_6_): *δ*=7.81 (d, *J*=8.1 Hz, 1H), 7.76 (d, *J*=8.1 Hz, 1H), 7.43 (apparent td, *J*=1.0, 8.1 Hz,1H), 7.35 (apparent td, *J*=1.0, 8.1 Hz, 1H), 6.91 (br s, 2H), 3.66 (m, 1H), 1.31-1.27 (m, 2H), 1.04-1.01 ppm (m, 2H); ^13^C NMR (125 MHz, DMSO-*d*_6_): *δ*=156.1 (s), 142.3 (s), 141.4 (s), 138.1 (s), 135.9 (s), 124.4 (d), 123.1 (d), 120.1 (d), 112.0 (d), 26.7 (d), 8.3 ppm (t); LC-MS: *m/z*: 242 [*M*+H]^+^, Rt = 4.67 min; HR LC-MS: *m/z* calcd for C_12_H_12_N_5_O: 242.1036 [*M*+H]^+^; found: 242.1038.

### 4-(1-Benzyl-1*H*-benzoimidazol-2-yl)-1,2,5-oxadiazole-3-ylamine (15d)

The benzimidazole (14d) (249 mg, 1.00 mmol) was diluted in MeOH (3 mL) and aqueous HCl (6 M, 1 mL). Solid NaNO_2_ (83 mg, 1.2 mmol, 1.2 equiv.) was added portionwise over 10 min. After the addition, the reaction mixture was made basic with aqueous NaOH (6 M, 1 mL) and hydroxylamine (50% aqueous solution, 0.34 mL, 5.1 mmol, 5.10 equiv.) was added. The reaction mixture was heated to reflux for 5 h, cooled and filtered (H_2_O and MeOH). Purification of the resulting solid by chromatography (SiO_2_, petroleum spirit/EtOAc: 70/30) afforded the title compound as a white solid (124 mg, 43%). ^1^H NMR (500 MHz, DMSO-*d*_6_): *δ*=7.87 (d, *J*=7.0 Hz, 1H), 7.72 (d, *J*=7.0 Hz, 1H), 7.41 (ddd, *J*=1.2, 7.1, 8.3 Hz, 1H), 7.37 (ddd, *J*=1.2, 7.1, 8.3 Hz, 1H), 7.30 (m, 2H), 7.24 (m, 1H), 7.15 (m, 2H), 7.03 (br s, 2H), 5.97 ppm (s, 2H); ^13^C NMR (125 MHz, DMSO-*d*_6_): *δ*=156.1 (s), 141.7 (s), 140.6 (s), 138.1 (s), 136.4 (s), 135.3 (s), 128.7 (2d), 127.5 (d), 126.5 (2d), 124.8 (d), 123.4 (d), 120.1 (d), 111.5 (d), 48.0 ppm (t); LC-MS: *m/z*: 292 [*M*+H]^+^, Rt = 5.07 min; HR LC-MS: *m/z* calcd for C_16_H_14_N_5_O: 292.1193 [*M*+H]^+^; found: 292.1199.

### 4-(1-Cyclopropylmethyl-1*H*-benzoimidazol-2-yl)-1,2,5-oxadiazole-3-ylamine (15e)

NaH (30 mg, 0.765 mmol) was added to a stirred solution of 2-aminoxadiazole benzimidazole (0.140 g, 0.696 mmol), in DMF (10 mL) at room temperature. The mixture was stirred at room temperature for 45 min, followed by the drop-wise addition of (bromomethyl)cyclopropane (0.188 g, 1.392 mmol) dissolved in DMF (1 mL). This mixture was heated at 65 °C for 18 h. After cooling to room temperature, the excess DMF was removed under reduced pressure and the resulting mixture was diluted with diethyl ether (50 mL). The organic mixture was washed with water and brine before drying over MgSO_4_. The excess solvent was removed under reduced pressure and the residue purified by flash chromatography (SiO_2_; EtOAc:hexane; 1:4) to afford title compound 15e (86 mg, 46%) as a white solid. ^1^H NMR (500 MHz, DMSO-*d*_6_): *δ*=7.82 (m, 2H), 7.43 (m, 1H), 7.36 (m, 1H), 6.99 (s, 2H), 4.59 (d, *J*=7.0 Hz, 2H), 1.36 (m, 1H), 0.48 ppm (m, 4H); ^13^C NMR (125 MHz, DMSO-*d*_6_): *δ*=156.2, 141.7, 140.2, 138.2, 135.4, 124.6, 123.1, 119.9, 111.5, 48.8, 11.3, 3.4 ppm; LC-MS: *m/z*: 256.11 [*M*+H]^+^, Rt = 2.90 min; HR LC-MS: *m/z* calcd for C_13_H_13_N_5_O: 256.2752 [*M*+H]^+^; found: 256.1194.

### *N*-[4-(1-Cyclopropyl-1*H*-benzoimidazol->2-yl)-1,2,5-oxadiazole-3-yl]-acetamide (16)

A mixture of amino-oxadiazole (15c) (50 mg, 0.207 mmol) and acetyl chloride (0.5 mL, 7.03 mmol) was stirred at room temperature for 2 h. The reaction mixture was partitioned between EtOAc and saturated aqueous sodium bicarbonate solution (200 mL). After separation, the organic phase was washed with brine, dried over MgSO_4_ and evaporated under vacuum. The crude product was purified by flash chromatography (SiO_2_, petroleum spirit/EtOAc, gradient: 80/20 to 70/30) to give 41 mg (70%) of the title compound as a white solid. ^1^H NMR (500 MHz, CDCl_3_): *δ*=11.07 (s, 1H), 7.84 (d, *J*=8.1 Hz, 1H), 7.72 (d, *J*=8.1 Hz, 1H), 7.60 (td, *J*=7.2, 1.0 Hz, 1H), 7.42 (td, *J*=7.2, 1.0 Hz, 1H), 3.77–3.45 (m, 1H), 2.46 (s, 3H), 1.45 (q, *J*=6.7 Hz, 2H), 1.20–1.04 ppm (m, 2H); ^13^C NMR (125 MHz, DMSO-*d*_6_): *δ*=168.3 (s), 150.1 (s), 141.6 (s), 141.2 (s), 135.9 (s), 124.4 (d), 123.1 (d), 120.2 (d), 111.9 (d), 99.5 (s), 26.0 (d), 23.3 (t), 7.7 ppm (t); LC-MS: *m/z*: 242 [*M*–(CH3C=O)+H]^+^, Rt = 2.41 min; HR LC-MS: *m/z* calcd for C_14_H_14_N_5_O_2_: 284.1142 [*M*+H]^+^; found: 284.1143.

### 4-[1-Ethyl-6-(3-methanesulfonyl-phenoxy)-1*H*-imidazo[4,5-c]pyridin-2-yl]-1,2,5-oxadiazole-3-ylamine (21b)

A glass tube (evacuated and back-filled with Ar) was charged with Fe(acac)_3_ (16.5 mg, 0.047 mmol), Cu powder (1.0 mg, 0.016 mmol), methyl-(3-hydroxyphenyl)sulfone (40 mg, 0.23 mmol), and Cs_2_CO_3_ (102 mg, 0.31 mmol). Bromoazabenzimidazole 21a (50 mg, 0.156 mmol) was added under Ar followed by anhydrous DMF (1.5 mL). The tube was sealed under Ar, and the mixture was heated to 150 °C under microwave irradiation for 30 min. After cooling to room temperature, the mixture was diluted with dichloromethane and filtered. The filtrate was concentrated *in vacuo* and partitioned between water and EtOAc. After separation, the water phase was extracted with EtOAc and the organics were combined, washed twice with water, dried over MgSO_4_ and concentrated. Purification of the residue by flash chromatography on silica gel (EtOAc/petroleum spirit, gradient: 40/60 to 70/30) afforded 21b (12 mg, 25%) as a white solid (13 mg, 20%). ^1^H NMR (500 MHz, DMSO-*d*_6_): *δ*=8.79 (s, 1H), 7.75 (apparent dt, *J*=1.2, 7.9 Hz, 1H), 7.70 (apparent t, *J*=7.9 Hz, 1H), 7.67 (s, 1H), 7.63 (apparent t, *J*=2.0 Hz, 1H), 7.50 (ddd, *J*=1.0, 2.3, 8.0 Hz, 1H), 6.92 (s, 2H), 4.70 (q, *J*=7.1 Hz, 2H), 3.25 (s, 3H), 1.41 ppm (t, *J*=7.1 Hz, 3H); ^13^C NMR (125 MHz, DMSO-*d*_6_): *δ*=158.2 (s), 156.1 (s), 155.6 (s), 143.1 (s), 143.0 (s), 142.2 (s), 140.0 (d), 137.8 (s), 137.1 (s), 130.9 (d), 125.3 (d), 122.1 (d), 118.2 (d), 93.7 (d), 43.3 (q), 40.6 (t), 14.6 ppm (q); LC-MS: *m/z*: 401 [*M*+H]^+^, Rt = 2.27 min; HR LC-MS: *m/z* calcd for C_17_H_17_N_6_O_4_S: 401.1026 [*M*+H]^+^; found: 401.1026.

### 4-(1-Ethyl-6-phenyl-1*H*-imidazo[4,5-c]pyridin-2-yl)-1,2,5-oxadiazole-3-ylamine (21c)

PhB(OH)_2_ (30 mg, 0.24 mmol), 1 M aqueous NaOH (0.48 mL, 0.48 mmol) and Pd(PPh_3_)_4_ (9.3 mg, 0.008 mmol) were added to a solution of 21a (50 mg, 0.16 mmol) in dimethoxyethane (DME, 2 mL). The reaction mixture was heated in a microwave reactor at 140 °C for 1.5 h. The reaction mixture was then partitioned between saturated aqueous NH_4_Cl solution and EtOAc. After separation, the organic phase was washed with brine, dried over MgSO_4_ and evaporated under vacuum. The residue was first purified by ion exchange chromatography on SCX-II acidic resin (2 g) eluting with MeOH then a 2 M solution of NH_3_ in MeOH. After concentration, the resulting solid was purified on a small pad of silica gel (EtOAc/petroleum spirit, gradient: 30/70 to 50/50) to afford 21c (29 mg, 59%) as a white solid. ^1^H NMR (500 MHz, DMSO-*d*_6_): *δ*=9.20 (s, 1H), 8.46 (s, 1H), 8.25 (d, *J*=7.3 Hz, 2H), 7.53 (dd, app t, *J*=7.7 Hz, 2H), 7.44 (dd, app t, *J*=7.3 Hz, 1H), 6.96 (s, 2H), 4.79 (q, *J*=7.1 Hz, 2H), 1.45 ppm (t, *J*=7.1 Hz, 3H); ^13^C NMR (125 MHz, DMSO-*d*_6_): *δ*=156.2 (s), 150.5 (s), 142.4 (s), 142.3 (d), 140.8 (s), 139.1 (s), 138.5 (s), 137.9 (s), 128.8 (d), 128.6 (d), 128.5 (d), 126.8 (d), 102.4 (d), 40.5 (t), 14.9 ppm (q); LC-MS: *m/z*: 307 [*M*+H]^+^, Rt = 2.23 min; HR LC-MS: *m/z* calcd for C_16_H_15_N_6_O: 308.1329 [*M*+H]^+^; found: 308.1337.

### 4-(1-Ethyl-6-morpholin-4-yl-1*H*-imidazo[4,5-c]pyridin-2-yl)-1,2,5-oxadiazole-3-ylamine (21d)

Morpholine (0.07 mL, 0.81 mmol) was added to a solution of 21a (25 mg, 0.081 mmol) in *N*-methylpyrrolidinone (0.5 mL). The reaction mixture was heated in a microwave reactor at 160 °C for 3 h. The mixture was cooled and concentrated. Purification of the crude by preparative TLC, eluting with EtOAc afforded 21d (13 mg, 51%) as a yellow solid. ^1^H NMR (500 MHz, DMSO-*d*_6_): *δ*=8.73 (s, 1H), 7.04 (s, 1H), 6.90 (s, 2H), 4.61 (q, *J*=7.1 Hz, 2H), 3.87-3.67 (m, 4H), 3.61-3.42 (m, 4H), 1.36 ppm (t, *J*=7.1 Hz, 3H); ^13^C NMR (125 MHz, DMSO-*d*_6_): *δ*=156.7 (s), 156.0 (s), 142.5 (s), 140.6 (s), 140.3 (d), 137.9 (s), 133.7 (s), 86.1 (d), 66.0 (t), 46.2 (t), 40.0 (t), 14.6 ppm (q); LC-MS: *m/z*: 316 [*M*+H]^+^, Rt = 2.50 min; HR LC-MS: *m/z* calcd for C_14_H_18_N_7_O_2_: 316.1516 [*M*+H]^+^; found: 316.1515.

### 4-(1-Ethyl-6-methyl-1*H*-imidazo[4,5-c]pyridin-2-yl)-1,2,5-oxadiazole-3-ylamine (21e)

MeB(OH)_2_ (29 mg, 0.48 mmol), 1 M aqueous NaOH (0.65 mL, 0.65 mmol) and Pd(PPh_3_)_4_ (9.3 mg, 0.008 mmol) were added to a solution of 21a (50 mg, 0.16 mmol) in DME (2 mL). The reaction mixture was heated in a microwave reactor at 140 °C for 2 h. The reaction mixture was then partitioned between saturated aqueous NH_4_Cl solution and EtOAc. After separation, the organic phase was washed with brine, dried over MgSO_4_ and evaporated under vacuum. Purification of the residue by flash chromatography (SiO_2_, EtOAc) afforded 21e (12 mg, 25%) as a white solid. ^1^H NMR (500 MHz, DMSO-*d*_6_): *δ*=8.99 (s, 1H), 7.70 (s, 1H), 6.93 (s, 2H), 4.66 (q, *J*=7.2 Hz, 2H), 2.63 (s, 3H), 1.39 ppm (t, *J*=7.2 Hz, 3H); ^13^C NMR (125 MHz, DMSO-*d*_6_): *δ*=156.1 (s), 151.7 (s), 141.8 (d), 141.5 (s), 140.2 (s), 137.9 (s), 137.3 (s), 104.5 (d), 40.3 (t), 24.3 (t), 14.7 ppm (q); LC-MS: *m/z*: 245 [*M*+H]^+^, Rt = 1.73 min; HR LC-MS: *m/z* calcd for C_11_H_13_N_6_O: 245.1145 [*M*+H]^+^; found: 245.1151.

### Crystallisation and structure determination

Crystallisation of the PKA-S6K1 chimera was performed using the same conditions as previously reported for the PKA/PKB chimera [21]. Prior to crystallisation, the purified protein was concentrated to 18 mg/mL in crystallisation buffer consisting of 25 mM MES-Bis/Tris pH 6.5, 75 mM LiCl, 0.1 mM EDTA, 1 mM octanoyl-*N*-methylglucamide and 1 mM DTT and containing 1 mM PKA-inhibitor peptide PKI (residues 5-24, Sigma-Aldrich) or an in-house synthesised peptide corresponding to residues 5-22 of the PKA-inhibitor peptide, but terminating with an amide at the *C*-terminus. Crystals of the PKA-S6K1/PKI staurosporine ternary complex were obtained by streak-seeding into fresh hanging drops containing the same protein and reservoir solutions as used for the crystallisation of the binary PKA-S6K1/PKI complex, with the addition of 0.5 mM staurosporine. All other protein-inhibitor complexes were obtained by soaking experiments of selected inhibitors at a final concentration of 1 mM as previously described for PKA [21]. Crystals were briefly transferred to a cryoprotectant solution containing 22.5% (*v/v*) 2-methyl-2,4-pentanediol, 25 mM MES-Bis/Tris pH 6.5, 0.1 mM EDTA and 1 mM DTT, prior to flash-cooling in liquid nitrogen. The data for the binary PKA-S6K1/PKI complex were collected at beamline ID14EH2 at the ESRF (Grenoble, France). Data for the ternary PKA-S6K1/PKI/inhibitor complexes were collected at the following beamlines: staurosporine- and compound 1-bound complexes were collected at beamline I02 of the Diamond Light Source (Oxfordshire, UK) and the data for the protein-inhibitor complexes with compounds 15e, 21a, and 21e were collected at beamline ID14EH4 at the ESRF. All data sets were integrated, merged and scaled using the programs iMosflm [36], Pointless and Aimless [37, 38] from the CCP4 suite [39] (see Supplementary Table S1 for data collection and refinement statistics). The PKA-S6K1/PKI structure was solved by molecular replacement using PHASER [40] with a PKA structure (PDB code: 2GFC) as a search model, after removal of all water molecules and truncation of the 5 mutated amino acids to alanine. All protein-inhibitor structures were solved by molecular replacement with the PKA-S6K1/PKI structure as a search model after removal of all water molecules. All structures were manually rebuilt in COOT [41] and refined with BUSTER [42] in iterative cycles. Ligand restraints were generated with Grade [43] and Mogul [44]. The quality of the structures was assessed with MOLPROBITY [45].

### Cellular assays

The human bladder carcinoma cell line HCV29 was obtained from Dr Richard Lamb (University of Liverpool, UK). This cell line has elevated mTORC1 and S6K activity through loss of TSC1 [46] as a result of a missense mutation and TSC2 loss that was confirmed by immunoblotting (data not shown). Cells were cultured in RPMI (Invitrogen, Life Technologies Ltd, Paisley, UK) containing 2 mmol/L glutamine (Invitrogen), and supplemented with non-essential amino acids (Invitrogen) 10% fetal bovine serum (PAA Laboratories Ltd, Yeovil, UK) in 5% CO_2_ in air at 37 °C. GI_50_ values (concentrations) causing 50% inhibition of proliferation for tumour cells were determined using a sulforhodamine B assay. Electrochemiluminescent immunoassays (Meso Scale Discovery, Rockville, MD, USA) measuring the inhibition of phosphorylation of RPS6-ser235/236, AKT-Thr308, AKT-Ser473, GSK3b-Ser9 and S6K-Ser421/424 were carried out as described in Raynaud *et al*. [47].

## Supplementary Tables


