# Supplementary material for: The discovery of potent ribosomal S6 kinase inhibitors by high-throughput screening and structure-guided drug design
Source: Oncotarget. 2013 Aug 25;4(10):1647–61. doi: 10.18632/oncotarget.1255 (PMC3858552; doi:10.18632/oncotarget.1255)
Supplement: Supplementary file 1 [file oncotarget-04-1647-s001.docx]

**The discovery of potent ribosomal S6 kinase inhibitors by high-throughput screening and structure-guided drug design**

Sylvain Couty^1,§^, Isaac M. Westwood^1,2,§^ Andrew Kalusa^1^, Celine Cano^3^, Jon Travers^1^, Kathy Boxall^1^, Chiau Ling Chow^1,2^, Sam Burns^1^, Jessica Schmitt^1^, Lisa Pickard^1^, Caterina Barillari^1,2^, Craig McAndrew^1^, Paul A. Clarke^1^, Spiros Linardopoulos^1^, Roger J. Griffin^3^, G. Wynne Aherne^1^, Florence I. Raynaud^1^, Paul Workman^1^, Keith Jones^1^*, Rob L.M. van Montfort^1,2^*

^1^ Cancer Research UK Cancer Therapeutics Unit, The Institute of Cancer Research, London SM2 5NG, UK

^2^ Division of Structural Biology, The Institute of Cancer Research, London SW3 6JB, UK

^3^ Northern Cancer Centre, Northern Institute for Cancer Research, School of Chemistry, Bedson Building, Newcastle University, Newcastle upon Tyne, NE1 7RU, UK

**Supplementary Information**

**Contents:**

**1) Details of the synthesis and characterisation of the precursors of oxadiazole-substituted benzimidazoles and azabenzimidazoles and synthesis of compounds 20a-g.**

**2) Protein production and purification**

**3) IC_50_ determination of screening hits using a DELFIA assay**

**4) IC_50_ determination of follow-up compounds hits using a mobility shift assay**

**5) Crystallographic data collection and refinement statistics.**

**1) Details of the synthesis and characterisation of oxadiazole-substituted benzimidazoles and azabenzimidazoles**

*N-Methylbenzene-1,2-diamine (****13a****)*


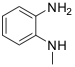


Anhydrous KF (1.16 g, 20.0 mmol) and K_2_CO_3_ (2.76 g, 20.0 mmol, 1 equiv.) were added to a mixture of 2-fluoronitrobenzene (2.1 mL, 20 mmol, 1 equiv.) and methylamine (10 mL, 2M solution in THF, 20 mmol, 1 equiv.) in a sealed tube. The reaction mixture was stirred at 80 °C for 0.5 h, cooled and treated with water and CH_2_Cl_2_. The organic layer was washed with 10% HCl and brine, dried over MgSO_4_ and evaporated under vacuum. The residue was dissolved in MeOH (100 mL) and stirred overnight at room temperature in the presence of Pd(10%)/C (1.06 g, 1.00 mmol, 0.05 equiv.). The reaction mixture was filtered on celite (MeOH) and evaporated under vacuum. The residue was purified by flash chromatography (SiO_2_, petroleum spirit/EtOAc, gradient: 70/30 to 50/50) to give 1.9 g (77%) of the title compound as a brown oil. ^1^H NMR (500 MHz, CDCl_3_): *δ*=6.87 (ddd, apparent td, *J*=1.8, 7.5 Hz, 1H), 6.73 (dd, *J*=1.8, 7.5 Hz, 1H), 6.71-6.66 (m, 2H), 3.34 (br s, 3H), 2.87 ppm (s, 3H); ^13^C NMR (125 MHz, CDCl_3_): *δ=*139.0 (s), 134.0 (s), 120.8 (d), 118.4 (d), 116.3 (d), 111.0 (d), 31.0 ppm (q).

*N-Ethylbenzene-1,2-diamine (****13b****)*


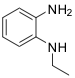


Anhydrous KF (1.16 g, 20.0 mmol) and K_2_CO_3_ (2.76 g, 20.0 mmol, 1 equiv.) were added to a mixture of 2-fluoronitrobenzene (2.1 mL, 20 mmol, 1 equiv.) and ethylamine (35 mL, 2 M solution in THF, 70 mmol, 3.5 equiv.) in a sealed tube. The reaction mixture was stirred at 90 °C for 1 h, cooled and treated with water and CH_2_Cl_2_. The organic layer was washed with 10% HCl and brine, dried over MgSO_4_ and evaporated under vacuum. The residue was dissolved in MeOH (100 mL) and stirred overnight at room temperature in the presence of Pd(10%)/C (1.06 g, 1.00 mmol, 0.05 equiv.). The reaction mixture was filtered on celite (MeOH) and evaporated under vacuum. The residue was purified by filtration (SiO_2_, petroleum spirit/EtOAc: 80/20) to give 2.29 g (84%) of the title compound as a dark oil. ^1^H NMR (500 MHz, CDCl_3_): *δ*=6.83 (ddd, apparent td, *J*=1.6, 7.5 Hz, 1H), 6.72 (dd, *J*=1.6, 7.5 Hz, 1H), 6.69-6.61 (m, 2H), 3.29 (br s, 3H), 3.16 (q, *J*=7.1 Hz, 2H), 1.31 ppm (t, *J*=7.1 Hz, 3H); ^13^C NMR (125 MHz, CDCl_3_): *δ*=137.8 (s), 133.9 (s), 121.0 (d), 118.9 (d), 116.8 (d), 112.3 (d), 38.9 (t), 14.8 ppm (q).

*N-Benzylbenzene-1,2-diamine (****13d****)*


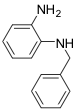


Anhydrous KF (209 mg, 3.60 mmol) and K_2_CO_3_ (497 mg, 3.60 mmol, 1 equiv.) were added to a mixture of 2-fluoronitrobenzene (380 μL, 3.60 mmol, 1 equiv.) and benzylamine (396 μL, 3.60 mmol, 1 equiv.) in a glass vial. After irradiation under microwave (100 °C, 15 min), the reaction mixture was cooled and treated with water and CH_2_Cl_2_. The organic layer was washed with 10% HCl and brine, dried over MgSO_4_ and evaporated under vacuum. The residue was dissolved in EtOH (70 mL) and stirred overnight at reflux in the presence of SnCl_2_·2H_2_O (9.33 g, 54.0 mmol, 15 equiv.). The reaction mixture was partitioned between EtOAc (200 mL) and saturated aqueous NaHCO_3_ (200 mL) solution. After separation, the organic phase was washed with brine, dried over MgSO_4_ and evaporated under vacuum to give 617 mg (86%) of the title compound as a dark oil. ^1^H NMR (500 MHz, CDCl_3_): *δ*=7.44-7.42 (m, 2H), 7.41-7.36 (m, 2H), 7.31 (m, 1H), 6.84 (ddd, apparent td, *J*=1.7, 7.5 Hz, 1H), 6.77 (dd, *J=*1.7, 7.5 Hz, 1H), 6.75-6.70 (m, 2H), 4.35 (s, 2H), 3.49 ppm (br s, 3H); ^13^C NMR (125 MHz, CDCl_3_): *δ*=139.5 (s), 137.8 (s), 134.2 (s), 128.6 (2d), 127.8 (2d), 127.3 (d), 120.8 (d), 118.9 (d), 116.6 (d), 112.1 (d), 48.7 ppm (t).

*(1-Methyl-1H-benzimidazol-2-yl)-acetonitrile (****14a****)*


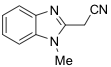


*N*-Methylbenzene-1,2-diamine (13a) (540 mg, 4.42 mmol) and ethyl cyanoacetate (1.90 mL, 17.7 mmol, 4 equiv.) were combined in a sealed tube and heated to 195 °C for 2 h. The reaction mixture was cooled, concentrated and purified by flash chromatography (SiO_2_, petroleum spirit/EtOAc, gradient: 60/40 to 40/60) to give 1.25 g (70%) of the title compound as a brown solid. ^1^H NMR (500 MHz, DMSO-*d_6_*): *δ*=7.63 (d, *J=*7.9 Hz, 1H), 7.54 (d, *J*=7.9 Hz, 1H), 7.27 (dd, apparent t, *J*=7.3 Hz, 1H), 7.21 (dd, apparent t, *J*=7.3 Hz, 1H), 4.51 (s, 2H) 3.76 ppm (s, 3H); ^13^C NMR (125 MHz, DMSO-*d_6_*): *δ=*145.6 (s), 141.7 (s), 135.9 (s), 122.4 (d), 121.8 (d), 118.8 (d), 116.2 (s), 110.1 (d), 29.8 (q), 17.3 ppm (t); LC-MS: *m/z:* 172 [*M*+H]^+^, Rt = 2.05 min; HR LC-MS: calcd for C_10_H_10_N_3_: 172.0869 [*M*+H]^+^; found: 172.0866.

*(1-Ethyl-1H-benzimidazol-2-yl)-acetonitrile (****14b****)*


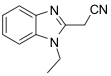


*N*-Ethylbenzene-1,2-diamine(13b) (1.00 g, 7.34 mmol) and ethyl cyanoacetate (3.10 mL, 29.4 mmol, 4 equiv.) were combined in a sealed tube and heated to 195 °C for 3 h. The reaction mixture was cooled, concentrated and purified by flash chromatography (SiO_2_, petroleum spirit/EtOAc, gradient: 60/40 to 40/60) to give 925 mg (68%) of the title compound as a brown solid. ^1^H NMR (500 MHz, DMSO-*d_6_*): *δ*=7.64 (d, *J*=7.9 Hz, 1H), 7.57 (d, *J*=7.9 Hz, 1H), 7.27 (ddd, apparent td, *J*=1.2, 7.9 Hz, 1H), 7.22 (ddd, apparent td, *J*=1.2, 7.9 Hz, 1H), 4.55 (s, 2H), 4.25 (q, *J*=7.2 Hz, 2H), 1.32 ppm (t, *J*=7.2 Hz, 3H); ^13^C NMR (125 MHz, DMSO-*d_6_*): 144.9 (s), 142.0 (s), 134.8 (s), 122.5 (d), 121.8 (d), 118.9 (d), 116.3 (s), 110.2 (d), 38.2 (t), 17.3 (t), 14.6 ppm (q); LC-MS: *m/z*: 186 [*M*+H]^+^, Rt = 2.67 min; HR LC-MS: *m/z* calcd for C_11_H_12_N_3_: 186.1026 [*M*+H]^+^; found: 186.1029.

*(1-Benzyl-1H-benzimidazol-2-yl)-acetonitrile (****14d****)*


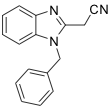


*N*-Benzylbenzene-1,2-diamine **(13d)** (510 mg, 2.57 mmol) and ethyl cyanoacetate (1.10 mL, 10.3 mmol, 4 equiv.) were combined in a sealed tube and heated to 195 °C for 2 h. The reaction mixture was cooled, concentrated and purified by flash chromatography (SiO_2_, petroleum spirit/EtOAc: 70/30) to give 274 mg (43%) of the title compound as a yellow solid. ^1^H NMR (500 MHz, DMSO-*d_6_*): δ=7.67 (m, 1H), 7.46 (m, 1H), 7.36-7.26 (m, 3H), 7.22 (m, 2H), 7.17 (apparent br s, 1H), 7.16 (apparent br s, 1H), 5.51 (s, 2H), 4.53 ppm (s, 2H). ^13^C NMR (125 MHz, DMSO-*d_6_*): 145.5 (s), 142.0 (s), 136.2 (s), 135.3 (s), 128.7 (2d), 127.7 (d), 126.8 (2d), 122.7 (d), 122.1 (d), 119.0 (d), 116.1 (s), 110.7 (d), 46.5 (t), 17.5 ppm (t); LC-MS: *m/z*: 248 [*M*+H]^+^, Rt = 4.04 min; HR LC-MS: *m/z* calcd for C_16_H_14_N_3_: 248.1182 [*M*+H]^+^; found: 248.1183.

*(1-Cyclopropyl-1H-benzimidazol-2-yl)-acetonitrile (****14c****)*


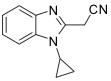


Anhydrous KF (335 mg, 5.77 mmol) and K_2_CO_3_ (797 mg, 5.77 mmol, 1 equiv.) were added to a mixture of 2-fluoronitrobenzene (610 μL, 5.77 mmol, 1 equiv.) and cyclopropylamine (1.2 mL, 17.3 mmol, 3 equiv.) in a sealed tube. The reaction mixture was stirred at 50 °C for 2 h, cooled and treated with water and ethyl acetate. The organic layer was washed with 10% HCl and brine, dried over MgSO_4_ and evaporated under vacuum. The residue was dissolved in ethanol (40 mL) and stirred 3.5 h at room temperature in the presence of Pd(10%)/C (310 mg, 0.29 mmol, 0.05 equiv.). The reaction mixture was filtered on celite (ethanol) and evaporated under vacuum. The residue was treated with ethyl cyanoacetate (2.46 mL, 23.1 mmol, 4 equiv.) in a sealed tube by heating at 195 °C for 2 h. The reaction mixture was cooled, concentrated and purified by flash chromatography (SiO_2_, petroleum spirit/EtOAc, gradient: 70/30 to 60/40) to give 490 mg (40%) of the title compound as a brown solid and 150 mg (12%) of a white solid intermediate. The latter compound was converted to the title compound by treatment with a crystal of CSA in toluene at reflux for 1 h (50% global yield). ^1^H NMR (500 MHz, CDCl_3_): δ=7.73 (apparent d, *J*=8.0 Hz, 1H), 7.55 (apparent d, *J*=8.0 Hz, 1H), 7.34-7.26 (m, 2H), 4.13 (s, 2H), 3.36 (m, 1H), 1.34 (m, 2H), 1.13 ppm (m, 2H); ^13^C NMR (125 MHz, CDCl_3_): δ=145.0 (s), 141.9 (s), 139.4 (s), 123.6 (d), 122.8 (d), 120.1 (d), 114.4 (s), 110.7 (d), 25.0 (d), 18.7 (t), 7.0 ppm (2t); LC-MS: *m/z*: 198 [*M*+H]^+^, Rt = 3.30 min; HR LC-MS: *m/z* calcd for C_12_H_12_N_3_: 198.1026 [*M*+H]^+^; found: 198.1032.

**
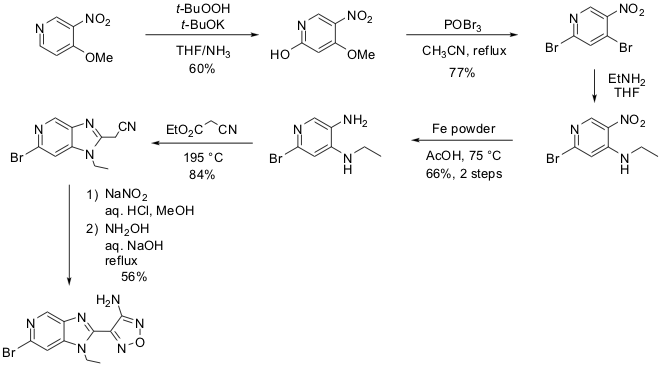
3-Amino-4-(6-Bromo-1-ethyl-1*H*-imidazo[4,5-c]pyridin-2-yl)-2,5-oxadiazole (21a)**

*4-Methoxy-5-nitro-1H-pyridin-2-one*


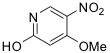


THF (25 mL) was cooled to -78 ºC and anhydrous NH3 (~80 mL) was condensed into THF. Potassium *t*-butoxide (9.1 g, 81.1 mmol) was added and the mixture was slowly warmed to -40 ºC. 4-Methoxy-3-nitropyridine (5 g, 32.4 mmol) was cooled to 0 ºC in THF (50 mL) and a solution of *t*-BuOOH (5 M in decane, 7.13 mL, 35.6 mmol) was added dropwise over 5 min. This solution was then added dropwise to the KO*t*-Bu solution prepared above over 40 min. After 90 min stirring at -35 ºC, the reaction mixture was carefully quenched with 30 mL of saturated aqueous NH_4_Cl solution. The mixture was allowed to vent and warm to room temperature overnight. Then the organics were concentrated, the residue made acidic with 6 M aqueous HCl solution and filtered (Et_2_O). Purification of the resulting solid by flash chromatography (SiO_2_, EtOAc/MeOH, gradient: 100/0 to 90/10) to afford 4-methoxy-5-nitro-1*H*-pyridin-2-one as a light yellow solid (3.31 g, 60%). ^1^H NMR (500 MHz, DMSO-*d_6_*): *δ*=12.18 (s, 1H), 8.50 (s, 1H), 5.89 (s, 1H), 3.86 ppm (s, 3H). ^13^C NMR (125 MHz, DMSO-*d_6_*): *δ*=162.8 (s), 160.9 (s), 139.7 (d), 125.6 (s), 96.5 (d), 56.7 ppm (q); LC-MS: *m/z*: 171 [*M*+H]^+^, Rt = 1.81 min.

*2,4-Dibromo-5-nitropyridine*


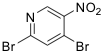


Phosphorous oxybromide (10.19 g, 35.56 mmol) was added to a suspension of 4-methoxy-5-nitro-1*H*-pyridin-2-one (3.025 g, 17.78 mmol) in acetonitrile (40 mL) at room temperature then heated to reflux for 3 h. The reaction mixture was cooled and carefully poured onto ice and saturated aqueous K2CO3 then extracted with ethyl acetate. The organic extracts were combined, washed with water and brine, dried (MgSO4), filtered and concentrated. Purification of the crude by filtration (SiO_2_, EtOAc/petroleum spirits 70/30) afforded 2,4-dibromo-5-nitropyridine as a light yellow solid (3.89 g, 77%). ^1^H NMR (500 MHz, DMSO-*d_6_*): *δ*=8.87 (s, 1H), 7.94 ppm (s, 1H); ^13^C NMR (125 MHz, DMSO-*d_6_*): *δ*=146.6 (d), 146.1 (s), 133.6 (d), 127.2 (s), 112.9 ppm (s); LC-MS (ESI): *m/z*: 282 [^81Br79Br^*M*+H]^+^, Rt = 4.08 min.

*2-bromo-N-ethyl-5-nitro-4-pyridinamine*


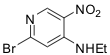


Ethylamine (2 M solution in THF, 11.5 mL, 23 mmol) was added dropwise over 1 h to a solution of 2,4-dibromo-5-nitro-pyridine (3.17 g, 11.24 mmol) in THF (30 mL). After 2 h at room temperature, the reaction mixture was poured into ice-cold H_2_O and extracted with ethyl acetate. The combined extracts were washed with brine, dried (MgSO4) and concentrated to give a yellow solid which was used without purification (2.5 g, 90%). ^1^H NMR (500 MHz, CDCl_3_): δ=8.96 (s, 1H), 8.05 (br s, 1H), 6.91 (s, 1H), 3.36 (qd, *J*=5.2, 7.2 Hz, 2H), 1.39 (t, *J*=7.2 Hz, 3H); ^13^C NMR (125 MHz, CDCl_3_): *δ*=149.1 (s), 149.0 (d), 147.6 (s), 110.6 (d), 100.0 (s), 37.8 (t), 13.9 ppm (q); LC-MS: *m/z*: 248 [^81Br^*M*+H]^+^, 246 [^79Br^*M*+H]^+^, Rt = 2.08 min.

*6-bromo-N4-ethyl-3,4-pyridinediamine*


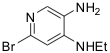


A solution 2-bromo-*N*-ethyl-5-nitro-4-pyridinamine (2 g, 8.13 mmol) in glacial acetic acid (40 mL) was added in portions over 30 min to a hot (75 ºC) suspension of iron powder (3.63 g, 65 mmol) in glacial acetic acid (70 mL). The reaction mixture was heated to 75 °C for an additional 2 h, then filtered through celite and concentrated. The residue was made basic with 6 M NaOH and extracted with ethyl acetate. The extracts were combined, washed with brine, dried (MgSO4) and concentrated to give the title compound as a dark oil which was used without purification (1.8 g, quantitative). ^1^H NMR (500 MHz, CDCl_3_): δ=7.61 (s, 1H), 6.57 (s, 1H), 4.21 (s, 1H), 3.17 (qd, *J*=5.2, 7.2 Hz, 2H), 2.95 (br s, 2H), 1.29 ppm (t, *J*=7.2 Hz, 3H); ^13^C NMR (125 MHz, CDCl_3_): δ=147.3 (s), 137.1 (d), 135.0 (s), 128.1 (s), 107.5 (d), 37.6 (t), 14.3 ppm (q); LC-MS: *m/z*: 218 [^81Br^*M*+H]^+^, 216 [^79Br^*M*+H]^+^, Rt = 1.20 min.

*(6-Bromo-1-ethyl-1H-imidazo[4,5-c]pyridin-2-yl)-acetonitrile*


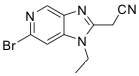


6-Bromo-*N*-ethyl-3,4-pyridinediamine (1.01 g, 4.67 mmol) and ethyl cyanoacetate (2 mL, 18.51 mmol) were combined in a sealed tube and heated to 195 °C for 30 min. The reaction mixture was cooled, concentrated and purified by flash chromatography (SiO_2_, petroleum spirit/EtOAc, gradient: 70/30 to 40/60) to give 1.03 g (84%) of the title compound as a yellow solid. ^1^H NMR (500 MHz, CDCl_3_): δ=8.82 (d, *J*=0.8 Hz, 1H), 7.53 (d, *J*=0.8 Hz, 1H), 4.26 (q, *J*=7.3 Hz, 2H), 4.10 (s, 2H), 1.54 ppm (t, *J*=7.3 Hz, 3H); ^13^C NMR (125 MHz, CDCl_3_): δ=145.6 (s), 142.6 (d), 142.1 (s), 139.4 (s), 133.8 (s), 113.4 (s), 108.7 (d), 39.8 (t), 18.1 (t), 14.8 ppm (q); LC-MS: *m/z*: 267 [^81Br^*M*+H]^+^, 265 [^79Br^*M*+H]^+^, Rt = 2.91 min; HR LC-MS: *m/z* calcd for C_10_H_10_BrN_4_: 265.0083 [^79Br^*M*+H]^+^; found: 265.0090.

*3-Amino-4-(6-bromo-1-ethyl-1H-imidazo[4,5-c]pyridin-2-yl)-1,2,5-oxadiazole (****21a****)*


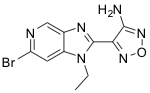


(6-Bromo-1-ethyl-1*H*-imidazo[4,5-*c*]pyridin-2-yl)-acetonitrile (490 mg, 1.85 mmol) was diluted in MeOH (2.5 mL) and aqueous HCl (6 M, 2.5 mL). Solid NaNO_2_ (153 mg, 1.93 mmol, 1.2 equiv) was added portionwise over 10 min. After the addition, the reaction mixture was made basic with aqueous NaOH (6 M, 2.5 mL) and hydroxylamine (50% aq., 0.63 mL, 8.30 mmol) was added. The reaction mixture was heated to reflux overnight, then cooled and filtered (H_2_O and MeOH). Purification of the resulting solid by filtration (SiO_2_, petroleum spirit/EtOAc: 60/40) afforded the title compound as a white solid (319 mg, 56%). ^1^H NMR (500 MHz, DMSO-*d_6_*): δ=8.93 (s, 1H), 8.25 (s, 1H), 6.92 (s, 2H), 4.68 (q, *J*=7.1 Hz, 2H), 1.38 ppm (t, *J*=7.1 Hz, 3H); ^13^C NMR (500 MHz, DMSO-*d_6_*): δ=156.2 (s), 143.3 (s), 142.5 (d), 141.9 (s), 139.0 (s), 137.8 (s), 135.5 (s), 101.1(d), 40.8 (t), 14.7 ppm (q); LC-MS : *m/z*: 311 [^81Br^*M*+H]^+^, Rt = 2.74 min ; HR LC-MS: *m/z* calcd for C_10_H_10_BrN_6_O: 309.0094 [^79Br^*M*+H]^+^; found: 309.0099.

**Synthesis of *C*-5 and *C*-7 substituted benzimidazole oxadiazoles**


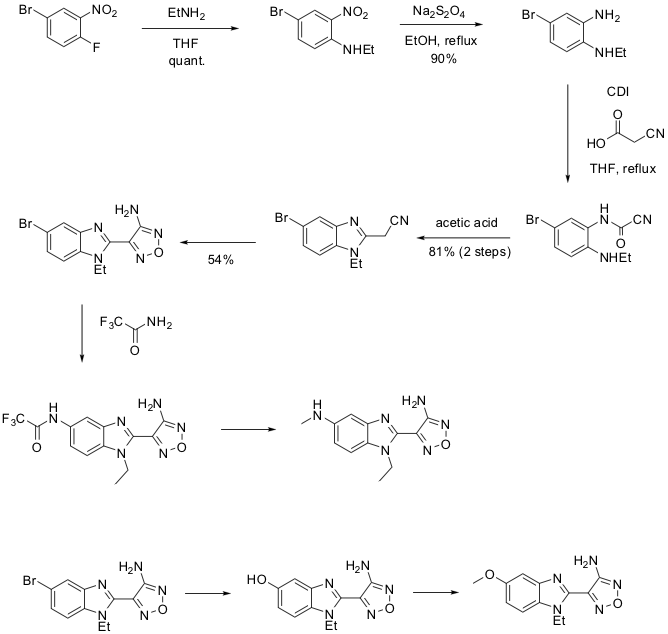


*(7-Bromo-1-ethyl-1H-benzimidazol-2yl)-acetonitrile*

In a similar manner (7-Bromo-1-ethyl-1*H*-benzimidazol-2yl)-acetonitrile was synthesised and used to prepare 7-substituted examples **20f** and **20g**.

*(4-Bromo-2-nitro-phenyl)-ethylamine*


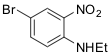


To a solution of **17a** (3.57 g, 16.23 mmol) in THF (100 mL) was added K_2_CO_3_ (2.92 g, 21.10 mmol) and EtNH_2_ (9.74 mL, 2 M solution in THF, 19.48 mmol). After 2 h at room temperature, the reaction mixture was partitioned between saturated aqueous NH_4_Cl solution and EtOAc. After separation, the organic phase was washed with brine, dried over MgSO_4_ and evaporated under vacuum to afford crude (4-bromo-2-nitrophenyl)-ethylamine (quantitative). (4-Bromo-2-nitrophenyl)-ethylamine was directly subjected to the next step without purification. ^1^H NMR (500 MHz, CDCl_3_): δ=8.31 (d, *J*=2.4 Hz, 2H), 7.49 (dd, *J*=2.4, 9.2 Hz, 1H), 6.76 (d, *J*=9.2 Hz, 1H), 3.40 (m, 2H), 1.37 ppm (t, *J*=7.2 Hz, 3H); ^13^C NMR (125 MHz, CDCl_3_): *δ*=144.4 (s), 138.9 (d), 132.1 (s), 129.0 (d), 115.5 (d), 106.2 (s), 37.8 (t), 14.3 ppm (q).

*4-Bromo-N^1^-ethyl-benzene-1,2-diamine (****18a****)*


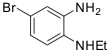


To a solution of (4-bromo-2-nitrophenyl)-ethylamine (3.98 g, 16.23 mmol) in EtOH (150 mL) was added a 1 M aqueous Na_2_O_4_S_2_ solution (114 mL, 114 mmol) and the resulting mixture was heated to reflux for 1 h. After concentration of the mixture, the residue was diluted with H_2_O (200 mL) and 2 M NaOH (30 mL). The organics were extracted with EtOAc (3x100 mL), washed with brine, filtrated and concentrated to give crude **18a** (3.218 g, 90%) as a yellow oil which slowly crystallised. Compound **18a** was directly subjected to the next step without purification. ^1^H NMR (500 MHz, CDCl_3_): δ=6.90 (dd, *J*=2.2, 8.4 Hz, 1H), 6.82 (d, *J*=2.2 Hz, 1H), 6.50 (d, *J*=8.4 Hz, 1H), 3.50-3.01 (br s, 3H), 3.11 (q, *J*=7.0 Hz, 2H), 1.29 ppm (t, *J*=7.0 Hz, 3H); LC-MS: *m/z*: 217 [^81Br^*M*+H]^+^, 215 [^79Br^*M*+H]^+^, Rt = 1.62 min.

*(5-Bromo-1-ethyl-1H-benzimidazol-2-yl)-acetonitrile (****19a****)*


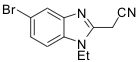


CDI (3.20 g, 19.72 mmol) was dissolved in THF (50 mL) with stirring at room temperature under N_2_ (g). Cyanoacetic acid (1.678 g, 19.72 mmol) was added carefully and the mixture was stirred for 10 min at room temperature until the evolution of CO_2_ ceased. Then **18a** (1.414 g, 6.57 mmol) was added and the reaction mixture was heated to reflux. After 2 h, more cyanoacetic acid (0.557 g, 6.57 mmol) and CDI (1.07 g, 6.57 mmol) were added. After 1 h, the reaction mixture was cooled and concentrated. The residue was dissolved in acetic acid (10 mL) and heated under reflux. After 2 h at reflux, the mixture was cooled, concentrated and carefully neutralised with saturated aqueous Na_2_CO_3_ solution. After addition of ethyl acetate and separation, the organic phase was washed with brine, dried over MgSO_4_ and evaporated under vacuum. Purification of the crude by column chromatography on silica gel (EtOAc/petroleum spirits: 60/40) gave **19a** (1.401 g, 5.30 mmol, 81% yield) as a yellow solid. ^1^H NMR (500 MHz, CDCl_3_): δ=7.89 (d, *J*=1.8 Hz, 1H), 7.43 (dd, *J*=1.8, 8.6 Hz, 1H), 7.24 (d, *J*=8.6 Hz, 1H), 4.25 (q, *J*=7.3 Hz, 2H), 4.06 (s, 2H), 1.50 ppm (t, *J*=7.3 Hz, 3H); ^13^C NMR (126 MHz, CDCl_3_): δ=143.9 (s), 143.8 (s), 139.9 (s), 126.6 (d), 123.1 (d), 116.0 (s), 114.2 (s), 110.9 (d), 39.5 (t), 18.0 (t), ppm 14.8 (q); LC-MS: *m/z*: 266 [^81Br^*M*+H]^+^, 264 [^79Br^*M*+H]^+^, Rt = 2.20 min; HR LC-MS: *m/z* calcd for C_11_H_11_BrN_3_: 264.0131 [^79Br^*M*+H]^+^; found: 264.0128.

*3-Amino-4-(5-bromo-1-ethyl-1H-benzimidazol-2-yl)-1,2,5-oxadiazole (****20a****)*


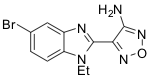


Benzimidazole **19a** (200 mg, 0.76 mmol) was diluted in MeOH (3 mL) and aqueous HCl (6 M, 3 mL). Solid NaNO_2_ (68 mg, 0.98 mmol) was added portionwise over 10 min. After the addition, the reaction mixture was made basic with the addition of aqueous NaOH (6 M, 3 mL) and hydroxylamine (50% aq. solution, 0.3 mL) was added. The reaction mixture was heated to reflux for 24 h. The reaction mixture was cooled and filtered (H_2_O and MeOH). Purification of the resulting solid by filtration [SiO_2_, petroleum spirits/Et_2_O (90/10) and petroleum spirits/EtOAc, gradient: 90/10 to 80/20) afforded the title compound as a white solid (125 mg, 54%). ^1^H NMR (500 MHz, DMSO-*d_6_*): δ=8.05 (s, 1H), 7.81 (d, *J*=8.6 Hz, 1H), 7.58 (dd, *J*=1.3, 8.6 Hz, 1H), 6.94 (s, 2H), 4.69 (q, *J*=7.1 Hz, 2H), 1.38 ppm (t, *J*=7.1 Hz, 3H); ^13^C NMR (126 MHz, DMSO-*d_6_*): δ=156.1 (s), 143.0 (s), 141.3 (s), 137.9 (s), 133.9 (s), 127.2 (d), 122.3 (d), 115.3 (s), 113.0 (d), 40.4 (t), 14.8 ppm (q); LC-MS: *m/z*: 310 [^81Br^*M*+H]^+^, 308 [^79Br^*M*+H]^+^, Rt = 2.73 min; HR LC-MS: *m/z* calcd for C_11_H_11_BrN_5_O: 308.0141 [^79Br^*M*+H]^+^; found: 308.0144.

*3-Amino-4-(1-ethyl-5-phenyl-1H-benzimidazol-2-yl)-1,2,5-oxadiazole (****20b****)*


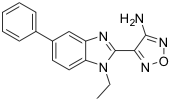


To a solution of **20a** (27 mg, 0.09 mmol) in DME (1.5 mL) was added PhB(OH)_2_ (16 mg, 0.13 mmol), 1 M aqueous NaOH (0.26 mL, 0.26 mmol) and Pd(PPh_3_)_4_ (5.0 mg, 0.004 mmol). The reaction mixture was heated in a microwave reactor at 140 ºC for 1.5 h. Then, the reaction mixture was partitioned between saturated aqueous NH_4_Cl solution and ethyl acetate. After separation, the organic phase was washed with brine, dried over MgSO_4_ and evaporated under vacuum. Purification of the crude by flash chromatography on silica gel (Hexane/Et_2_O 90/10 then Hexane/EtOAc 90/10) afforded **20b** (19 mg, 71%) as a white solid. ^1^H NMR (500 MHz, CDCl_3_): δ=8.03 (d, *J*=1.7 Hz, 1H), 7.69-7.65 (m, 3H), 7.55 (d, *J*=8.5 Hz, 1H), 7.48 (m, 2H), 7.37 (m, 1H), 5.93 (s, 2H), 4.77 (q, *J*=7.2 Hz, 2H), 1.54 ppm (t, *J*=7.2 Hz, 3H); ^13^C NMR (126 MHz, CDCl_3_): δ=156.2 (s), 143.3 (s), 141.5 (s), 141.3 (s), 138.5 (s), 137.3 (s), 134.7 (s), 129.1 (d), 127.6 (d), 127.3 (d), 124.8 (d), 119.0 (d), 110.4 (d), 40.9 (t), 15.2 ppm (q); LC-MS: *m/z*: 306 [*M*+H]^+^; Rt = 2.87 min; HR LC-MS: *m/z* calcd for C_17_H_16_N_5_O: 307.1378 [*M*+H]^+^, found 307.1385.

*3-Amino-4-(5-cyclopropyl-1-ethyl-1H-benzimidazol-2-yl)-1,2,5-oxadiazole (****20c****)*

.
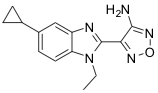


To a solution of **20a** (40 mg, 0.13 mmol) in DME (1.5 mL) was added cyclopropylboronic acid (65.4 mg, 0.39 mmol), 1 M aqueous NaOH (0.39 mL, 0.39 mmol) and Pd(PPh_3_)_4_ (7.5 mg, 0.006 mmol). The reaction mixture was heated in a microwave reactor at 140 ºC for 1.5 h. Then, the reaction mixture was partitioned between saturated aqueous NH_4_Cl solution and EtOAc. After separation, the organic phase was washed with brine, dried over MgSO_4_ and evaporated under vacuum. LC/MS of the crude indicated the presence of the target material contaminated with the debrominated starting material. This impurity could not be removed by chromatography. Eventually, 19 mg of the crude material was purified by semi-preparative HPLC (MeCN/H_2_O, 90/10; Nucleosil, length:250 mm, internal diameter:10 mm, Macheey-Nagel) to afford the title compound (5 mg, 14%) as a white solid. ^1^H NMR (500 MHz, CDCl_3_): δ=7.51 (s, 1H), 7.36 (d, *J=*8.4 Hz, 1H), 7.20 (dd, *J*=1.3, 8.4 Hz, 1H), 5.91 (s, 2H), 4.70 (q, *J*=7.2 Hz, 2H), 2.06 (m, 1H), 1.48 (t, *J*=7.2 Hz, 3H), 1.01 (m, 2H), 0.76 ppm (m, 2H); ^13^C NMR (126 MHz, CDCl_3_): δ=156.12 (s), 143.04 (s), 140.62 (s), 139.60 (s), 138.51 (s), 133.55 (s), 124.09 (d), 117.01 (d), 109.77 (d), 40.73 (t), 15.74 (d), 15.21 (q), 9.29 ppm (t); LC-MS: *m/z*: 270 [*M*+H]^+^; Rt = 2.75 min; HR LC-MS: *m/z* calcd for C_14_H_16_N_5_O: 271.1377 [*M*+H]^+^; found: 271.1381.

*3-Amino-4-(1-ethyl-5-hydroxy-1H-benzoimidazol-2-yl)-1,2,5-oxadiazole*


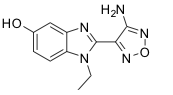


To a solution of **20a** (40 mg, 0.13 mmol) in THF (1.5 mL) at -78 °C was added *n*-BuLi (0.2 ml, 2 M solution in hexanes, 0.40 mmol, 3.1 equiv.). The resulting red solution was stirred for 5 min, then B(OMe)_3_ (0.05 mL, 0.43 mmol, 3.3 equiv) was added and the cooling bath was removed. After 3 h, 3 M aqueous NaOH (0.1 mL) and 30% (*w/w*) H_2_O_2_ (0.3 mL) were added and the mixture was stirred for 1.5 h. The reaction mixture was partitioned between EtOAc and 3 M aqueous HCl. After separation, the organic phase was washed with brine, dried over MgSO_4_ and evaporated under vacuum. Purification of the crude by chromatography on a Biotage column (Hexane/EtOAc gradient, 93/7 to 40/60) afforded 3-amino 4-(1-ethyl-5-hydroxy-1*H*-benzimidazo-2-yl)-1,2,5-oxadiazole (13 mg, 41%) as a white solid. ^1^H NMR (500 MHz, MeOD): δ=7.46 (d, *J*=8.8 Hz, 1H), 7.13 (d, *J*=2.2 Hz, 1H), 6.97 (dd, *J*=2.2, 8.8 Hz, 1H), 4.69 (q, *J*=7.2 Hz, 2H), 1.45 ppm (t, *J*=7.2 Hz, 3H); ^13^C NMR (126 MHz, MeOD): δ=157.7 (s), 155.6 (s), 144.8 (s), 141.6 (s), 139.7 (s), 130.6 (s), 116.2 (d), 111.9 (d), 105.2 (d), 41.4 (t), 15.4 ppm (q); LC-MS: *m/z*: 246 [*M*+H]^+^, Rt = 2.73 min; HR LC-MS: *m/z* calcd for C_11_H_12_N_5_O_2_: 246.0986[*M*+H]^+^; found: 246.0991.

*3-Amino-4-(1-ethyl-5-methoxy-1H-benzimidazol-2-yl)-1,2,5-oxadiazole (****20d****)*


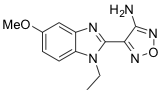


To a solution of 4-(1-ethyl-5-methoxy-1*H*-benzoimidazol-2-yl)-furazan-3-ylamine (13 mg, 0.05 mmol) in acetone (1.5 mL) was successively added K_2_CO_3_ (70 mg, 0.53 mmol, 10 equiv.) and an excess of MeI (0.1 mL, 1.60 mmol). The mixture was heated under reflux for 2 h, cooled and left at room temperature overnight. The reaction mixture was partitioned between ethyl acetate and saturated aqueous NH_4_Cl solution. After separation, the organic phase was washed with brine, dried over MgSO_4_ and evaporated under vacuum. Purification of the crude by chromatography on a Biotage column (Hexane/EtOAc gradient, 95/5 to 40/60) afforded **20d** (11 mg, 80%) as a white solid. ^1^H NMR (500 MHz, CDCl_3_): δ=7.36 (d, *J*=8.9 Hz, 1H), 7.26 (d, *J*=2.4 Hz, 1H), 7.07 (dd, *J*=2.4, 8.9 Hz, 1H), 5.91 (s, 2H), 4.69 (q, *J*=7.2 Hz, 2H), 3.89 (s, 3H), 1.49 ppm (t, *J*=7.2 Hz, 3H); ^13^C NMR (126 MHz, CDCl_3_): δ=157.1 (s), 156.1 (s), 143.5 (s), 140.6 (s), 138.5 (s), 129.9 (s), 115.7 (d), 110.6 (d), 102.0 (d), 56.0 (q), 40.8 (t), 15.3 ppm (q); LC-MS: *m/z*: 260 [*M*+H]^+^, Rt = 2.49 min; HR LC-MS: *m/z* calcd for C_12_H_14_N_5_O_2_: 260.1142 [*M*+H]^+^; found: 260.1147.

*3-Amino-4-(1-ethyl-5-trifluoroacetamido-1H-benzimidazol-2-yl)-1,2,5-oxadiazole*


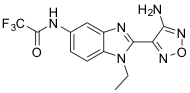


An oven-dried glass tube was charged with CuI (2.5 mg, 0.013 mmol), K_2_CO_3_ (36 mg, 0.26 mmol), and 4 Å MS (65 mg). The tube was evacuated and backfilled with Ar (g). Then **20a** (40 mg, 0.13 mmol), trifluoroacetamide (22 mg, 0.175 mmol), *N*,*N*′dimethylethylenediamine (3.25 μL, 0.026 mmol), and dioxane (1.5 mL) were added under Ar. The reaction mixture was stirred for 24 h at 80 °C. TLC monitoring showed only 50% conversion but the resulting suspension was cooled to room temperature and filtered through a pad of celite (EtOAc). The filtrate was concentrated, and the residue was purified by flash chromatography (Hexane/EtOAc, gradient: 90/10 to 70/30) to afford [2-(4-amino-furazan-3-yl)-1-ethyl-1*H*-benzoimidazol-5-yl]-methylamine (11 mg, 25%) as a white solid. ^1^H NMR (500 MHz, MeOD): δ=8.18 (d, *J*=1.8 Hz, 1H), 7.69 (d, *J*=8.8 Hz, 1H), 7.65 (dd, *J*=1.8, 8.8 Hz, 1H), 4.77 (q, *J*=7.2 Hz, 2H), 1.47 ppm (t, *J*=7.2 Hz, 3H); LC-MS: *m/z*: 341 [*M*+H]^+^, Rt = 2.39 min.

*3-Amino-4-(1-ethyl-5-methylamino-1H-benzimidazol-2-yl)-1,2,5-oxadiazole (****20e****)*


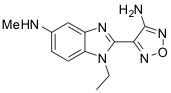


To a solution of *3-Amino-4-(1-ethyl-5-trifluoroacetamido-1H-benzimidazol-2-yl)-1,2,5-oxadiazole* (11 mg, 0.032 mmol) in acetone (1.5 mL) was added MeI (0.02 mL, 0.32 mmol, 10 equiv.) and powdered KOH (10 mg, 0.13 mmol, 4 equiv.). The reaction mixture was heated under reflux for 1 h then cooled down and concentrated under vacuum. The residue was dissolved in EtOH (3 mL) and treated with 5% aqueous NaOH (1 mL) at reflux. After 15 min, the mixture was partitioned between water and EtOAc. After separation, the organic phase was washed with brine, dried over MgSO_4_ and evaporated under vacuum. Purification of the crude by preparative TLC, eluting with Hexanes/EtOAc (80/20, 4 elutions) afforded **20e** (5.5 mg, 70%) as a green solid. ^1^H NMR (500 MHz, CDCl_3_): δ=7.27 (d, *J*=8.7 Hz, 1H), 6.94 (d, *J*=2.2 Hz, 1H), 6.80 (dd, *J*=2.2, 8.7 Hz, 1H), 5.92 (s, 2H), 4.66 (q, *J*=7.2 Hz, 2H), 2.91 (s, 3H), 1.47 ppm (t, *J*=7.2 Hz, 3H); ^13^C NMR (126 MHz, CDCl_3_): δ=156.10 (s), 146.81 (s), 144.22 (s), 139.79 (s), 138.59 (s), 128.53 (s), 114.79 (d), 110.53 (d), 100.26 (d), 40.65 (t), 31.63 (q), 15.29 ppm (q); LC-MS: *m/z*: 259 [*M*+H]^+^, Rt = 1.57 min; HR LC-MS: *m/z* calcd for C_12_H_15_N_6_O: 259.1302 [*M*+H]^+^; found: 259.1302.

*3*-*Amino*-*4-(7-bromo-1-ethyl-1H-benzimidazol-2-yl)-1,2,5-oxadiazole (****20f****)*


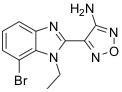


(7-Bromo-1-ethyl-1H-benzimidazol-2-yl)-acetonitrile (150 mg, 0.56 mmol) was dissolved in MeOH (3 mL) and aqueous HCl (6 M, 3 mL). Solid NaNO_2_ (51 mg, 0.74 mmol,) was added portion-wise over 10 min. After the addition, the reaction mixture was made basic with the addition of aqueous NaOH (6 M, 3 mL) and hydroxylamine (50% aqueous solution, 0.3 mL) was added. The reaction mixture was heated to reflux for 24 h. The reaction mixture was cooled and filtered (H_2_O and MeOH). Purification of the resulting solid by filtration [SiO_2_, petroleum spirits/Et_2_O (90/10) and petroleum spirits/EtOAc, gradient: 90/10 to 80/20) afforded the title compound as a white solid (79 mg, 44%). ^1^H NMR (400 MHz, CDCl_3_): δ=7.35-7.17 (m, 3H), 4.27-4.11 (m, 2H), 1.53-1.34 ppm (m, 3H); ^13^C NMR (126 MHz, DMSO): δ=145.42, 142.42, 135.27, 122.97, 122.31, 119.44, 116.92, 110.72, 17.77, 15.14 ppm; LC-MS: *m/z*: 309.01 [*M*+H]^+^, Rt = 3.30 min; HR LC-MS: *m/z* calcd for C_11_H_11_BrN_5_O: 309.1340 [*M*+H]^+^; found: 309.0094.

*3-Amino-4-(7-(3-pyridinyl)-1-ethyl-1H-benzimidazol-2-yl)-1,2,5-oxadiazole (****20g****)*


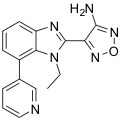


To a solution of **20f** (0.105 mg, 0.341 mmol) in DME (1.5 mL) was added 3-pyridinylboronic acid (54 mg, 0.443 mmol), 1 M aqueous NaOH (1.02 mL, 1.023 mmol) and Pd(PPh_3_)_4_ (16 mg, 0.013 mmol). The reaction mixture was heated in a microwave reactor at 140 ºC for 1.5 h. The reaction mixture was partitioned between saturated aqueous NH_4_Cl solution and ethyl acetate. After separation, the organic phase was washed with brine, dried over MgSO_4_ and evaporated under vacuum. Purification of the crude by flash chromatography on silica gel (Hexane/Et_2_O 90/10 then Hexane/EtOAc 90/10) afforded title compound **20g** (16 mg, 15%) as a white solid. ^1^H NMR (400 MHz, DMSO-*d_6_*): δ=8.84-8.65 (m, 2H), 7.93 (ddd, *J*=8.3, 3.9, 1.0 Hz, 2H), 7.61-7.34 (m, 3H), 7.11-6.96 (s, 2H), 4.11-3.91 (m, 2H), 0.92 ppm (t, *J*=7.7 Hz, 3H); ^13^C NMR (126 MHz, DMSO-*d_6_*): δ=156.78, 150.14, 137.07, 120.97, 115.38, 110.84, 99.88, 23.39, 15.14 ppm; LC-MS: *m/z*: 306.13 [*M*]^+^, Rt = 2.80 min; HR found 307.1306; C_16_H_15_N_6_O [M + H]^+^ requires 307.3220.

**2) Protein production and purification**

The His-tagged kinase domain of S6K1 (residues 1-421, catalogue number 14-486) used in the HTS was purchased from Upstate/Millipore (Merck Group, Darmstadt, Germany). GST-tagged kinase domain of S6K1 (residues 1-421, catalogue number 38944B) used in IC_50_ determinations was purchased from Invitrogen (catalogue number 38944B, Carlsbad CA, USA). The PKA-S6K1 chimera was created by introducing five point mutations into bovine PKAα (genbank accession # NM_174584) by gene synthesis. These mutations resulted in the following amino acid changes: F55Y, M121L, V124L, L174M, and Q182K. The synthesised gene was expressed as an *N*-terminal hexa-histidine tagged fusion protein in the BL21 (DE3) *Escherichia coli* strain (Merck4Biosciences, Merck Chemicals Ltd, Nottingham, UK) grown in Overnight Express Instant TB autoinduction media (Merck4Biosciences) at 37 °C and harvested after 16-18 h.

Cell pellets were resuspended in Talon buffer A consisting of 50 mM HEPES pH 7.0, 300 mM NaCl, and 10% *(v/v)* glycerol. Complete EDTA-free protease inhibitors (Roche Diagnostics, Burgess Hill, UK), Benzonase (Merck4Biosciences) were added at 12.5 U/mL and lysozyme at 1 mg/mL. Following sonication and clarification, the lysate was mixed with 4 mL Talon resin (Takara Bio Europe/Clontech Laboratories Inc., Mountain View, CA, USA) per litre of culture. The resin was washed with 5 column volumes (CV) of Talon buffer A, 20 CV of Talon Buffer B comprising 50 mM HEPES pH 7.4, 300 mM NaCl, 10% *(v/v)* glycerol) and 5 mM imidazole and eluted with Talon buffer C containing 50 mM HEPES pH 7.4, 300 mM NaCl, 10% *(v/v)* glycerol and 250 mM imidazole. The eluted fractions were supplemented with 1 mM EDTA, 1 mM DTT and 3.6 U/mg of Prescission Protease (GE Healthcare, Little Chalfront, UK) and incubated for 16 h at 4 °C to remove the *N*-terminal His-tag.

The cleaved eluted fractions were desalted against Resource-S buffer A, consisting of 25 mM Bis-Tris propane pH 8.5, and loaded on a 6 mL Resource S column (GE Healthcare). Bound protein was eluted with a gradient from 0 to 30% Resource-S buffer B comprising 25 mM Bis-Tris propane pH 8.5 and 1 M LiCl over 45 CV. Of the 3 major peaks eluted, fractions from the first peak were pooled and loaded on a Hiload 26/60 Superdex 75 preparative grade column (GE Healthcare) equilibrated in 50 mM HEPES pH 7.5, 200 mM NaCl and 10% *(v/v)* glycerol. Peak fractions were pooled and mixed with Talon resin (300 mL per litre culture) equilibrated with Talon buffer B. Following binding, the resin was washed with 20 CV of Talon buffer B additionally containing 5 mM imidazole. The flow through and wash fractions containing protein were pooled, supplemented with 1 mM EDTA and 1 mM DTT, concentrated and frozen prior to crystallisation.

**3) IC_50_ determination of screening hits using a DELFIA assay**

For primary screening hits, IC_50_ values were determined using a DELFIA^®^ assay that monitors phosphorylation of a S6 peptide using a specific phospho-antibody. The enzyme reaction was carried out in 96 well polypropylene plates (#650261; Greiner Bio-One, Frickenhausen, Germany).

For each compound, a 10mM stock was diluted in water resulting in a 1250 μM solution in 12.5% *(v/v)* DMSO. Compounds were manually diluted 1 in 3 in 12.5% *(v/v)* DMSO to give an eight point dilution curve in duplicate. Finally, 5 μL compound solution or 12.5% *(v/v)* DMSO was transferred to the assay plate resulting in compound assay concentrations of 0.12-250 μM.

Subsequently, 20 μL of reaction reagents were added to the assay plate resulting in final assay concentrations of 1 μM S6 peptide (biotin-KRRRLSSLRASTSKSESSQKI, synthesised by J. Metcalfe, ICR), 2 nM S6K1 (#14-486; Millipore, Billerica, MA, USA) and 30 μM ATP in assay buffer consisting of 40 mM HEPES pH 7.4, 10 mM MgCl_2_, 1 mM DTT and 0.02% *(v/v)* Tween 20. The reaction was incubated for an hour at 37 °C and stopped by the addition of 125 μL buffer comprising 40 mM EDTA, 0.05% *(v/v)* Tween 20, 0.1% *(w/v)* BSA in TBS (10x concentrate, Sigma-Aldrich, Gillingham, UK). A 100 μL aliquot of the reaction solution was transferred to a black neutravidin-coated plate (#15117; Perbio/Thermo Scientific, Cramlington, UK) and incubated for an hour on a Titertek shaker (Flow Laboratories, Sutton, UK) at room temperature. The plates were washed four times with WellWash4 wash buffer (Thermo Life Sciences Ltd, Basingstoke, UK) containing 25 mM Tris pH 8, 150 mM NaCl and 0.1% *(v/v)* Tween 20 and incubated for an hour with 100 μL of an antibody mix consisting of 0.05 nM of an anti-phospho S6 antibody (#2211; Cell Signalling Technology) and 0.18 μg/mL of an europium-labelled anti-rabbit IgG, (#AD0105; PerkinElmer Life Sciences, Waltham, MA, USA) diluted in DELFIA assay buffer (#4002-0010; PerkinElmer Life Sciences). The plates were washed a further four times with WellWash4 wash buffer (Thermo Life Sciences) before the addition of 100 μL Enhancement Solution (#4001-0010; PerkinElmer Life Sciences). The plate was read on an EnVision® 2103 Multilabel Reader (PerkinElmer Life Sciences) using a time-resolved measurement mode reading fluorescence at 615 nm. IC_50_ values were calculated from a four-parameter logistics fit of percentage inhibition versus concentration using GraphPad Prism 5 (GraphPad Software, Inc., CA, USA).

**4) IC_50_ determination using a mobility shift assay**

For all compounds from the benzimidazole oxadiazole/azabenzimidazole oxadiazole series, the IC_50_ values were determined using a microfluidic assay that monitors the separation of a phosphorylated product from its substrate. The assay was performed on an EZ Reader II (PerkinElmer Life Sciences, Waltham, MA, USA) using separation buffer (#760367; PerkinElmer Life Sciences) containing 500nM CR-8 (#760278; PerkinElmer Life Sciences). For each compound, a 10 mM stock concentration in 100% DMSO was used. Compounds were diluted manually 1 in 3 in 100% DMSO to give an eight point dilution curve before a second 10-fold dilution step in the assay buffer consisting of 40 mM HEPES (pH 7.5), 10 mM MgCl_2_, 1 mM DTT, 0.02% Tween20. Subsequently, 3 μL of the diluted compound solution was transferred into the assay plate to give a final concentration of 1.5% *(v/v)* DMSO and compounds in the range 0.5 nM-100 μM.

The enzyme reaction (total volume 20 μL) was carried out in 384-well polypropylene plates (#781280; Greiner Bio-One, Frickenhausen, Germany) containing 1.3nM of the catalytic domain of S6K1 (#PV3815; Life Technologies, NY USA), 1.5 μM fluorescently labeled peptide (FL-Peptide1, #760345; PerkinElmer Life Sciences, sequence: 5FAM-AKRRLSSLRA-CONH_2_), 1.5 μM ATP, either DMSO (1.5% *v/v*) or the test compound and assay buffer. The reaction was carried out for an hour at room temperature and stopped by the addition of 80 μL Separation buffer (#760367; PerkinElmer Life Sciences), containing 100 mM HEPES pH 7.3, 0.015% *(v/v)* Brij-35, 5% *(v/v)* DMSO, 0.1% *(v/v)* Coating reagent 3, 0.05 μM and 10 mM EDTA.

The plate was read on an EZ Reader II, using a 12-sipper chip (#760404; PerkinElmer Life Sciences) with instrument settings of -1.5 psi and 1750 ∆V. The percentage conversion of substrate to product was carried out using the EZ Reader II software package Reviewer. The percentage inhibition was calculated relative to blank wells (containing no enzyme and 1.5% (v/v) DMSO). IC_50_ values were calculated from a four-parameter logistics fit of percentage inhibition versus concentration using GraphPad Prism 5 (GraphPad Software, Inc., CA, USA).

**5) Table S1: Crystallographic data collection and refinement statistics.**

| Ligand | PKA-S6K1/PKI | Staurosporine | Compound **1** | Compound **15e** |
| --- | --- | --- | --- | --- |
| Crystals |  |  |  |  |
| Space group | P2_1_2_1_2_1_ | P2_1_2_1_2_1_ | P2_1_2_1_2_1_ | P2_1_2_1_2_1_ |
| Lattice constants |  |  |  |  |
| a (Å) | 71.72 | 69.43 | 70.44 | 72.56 |
| b (Å) | 75.2 | 72.71 | 73.38 | 75.85 |
| c (Å) | 80.12 | 77.09 | 77.34 | 80.38 |
| α, β, γ (°) | 90 | 90 | 90 | 90 |
|  |  |  |  |  |
| Data collection |  |  |  |  |
| Beamline | ESRF ID14EH2 | Diamond I02 | Diamond I02 | ESRF ID14EH4 |
| Wavelength (Å) | 0.933 | 0.9795 | 0.9795 | 0.9795 |
| Resolution range (Å) | 30.01-1.70 | 42.08-1.78 | 53.23-2.19 | 55.17-1.98 |
| (highest-resolution shell values) | (1.73-1.70) | (1.82-1.78) | (2.26-2.19) | (2.03-1.98) |
| Observations | 125313 (3227) | 125880 (6692) | 82601 (7070) | 122403 (8384) |
| Unique reflections | 39431 (1236) | 37804 (2115) | 21188 (1812) | 31577 (2196) |
| Completeness (%) | 82.5 (50.8) | 99.6 (99.9) | 99.6 (99.7) | 99.8 (100) |
| Multiplicity | 3.2 (2.6) | 3.3 (3.2) | 3.9 (3.9) | 3.9 (3.8) |
| R_merge_ (%) | 5.4 (19.2) | 6.4 (50.8) | 5.4 (35.3) | 5.9 (35.6) |
| I/σ(I) | 7.5 (3.7) | 6.3 (1.3) | 7.3 (2.1) | 8.7 (1.9) |
| Mean I/σ(I) | 11.4 (3.8) | 13.9 (2.1) | 15.6 (3.9) | 12.5 (3.3) |
| CC_1/2_^[a]^ | 0.996 (0.932) | 0.996 (0.725) | 0.996 (0.873) | 0.997 (0.827) |
| Average Mosaicity (º) | 1.24 | 0.39 | 0.6 | 0.65 |
|  |  |  |  |  |
| Refinement |  |  |  |  |
| No. of amino acids S6K-PKA | 338 | 340 | 341 | 338 |
| No. of amino acids PKI peptide | 18/18 | 18/18 | 18/18 | 19/20 |
| No. of water molecules | 596 | 379 | 178 | 377 |
| No. of Methanol molecules | - | - | - | 1 |
| No. of MPD molecules | - | - | 1 | - |
| No. of MEGA-8 molecules | - | 1 | - | - |
| No. of glycerol molecules | - | 1 | - | - |
| Ligand bound to active site |  | Staurosporine | Compound **1** | Compound **15e** |
| R-factor (%) | 15.6 | 17.8 | 18.3 | 15.7 |
| R_free_^a^ (%) | 18.7 | 21.5 | 24 | 19 |
|  |  |  |  |  |
| Ramachandran plot (%)^[b]^ |  |  |  |  |
| Favoured | 98.3 | 98 | 96.9 | 97.7 |
| Outliers | 0.3 | 0 | 0 | 0 |
|  |  |  |  |  |
| RMSD bonds | 0.01 | 0.01 | 0.01 | 0.01 |
| RMSD angles | 0.94 | 0.96 | 1.02 | 0.94 |

**Table S1 continued: Crystallographic data collection and refinement statistics.**

| Ligand | Compound **21a** | Compound **21e** |
| --- | --- | --- |
| Crystals |  |  |
| Space group | P2_1_2_1_2_1_ | P2_1_2_1_2_1_ |
| Lattice constants |  |  |
| a (Å) | 72.36 | 72.14 |
| b (Å) | 75.1 | 75.09 |
| c (Å) | 80.27 | 80.19 |
| α, β, γ (°) | 90 | 90 |
|  |  |  |
| Data collection |  |  |
| Beamline | ESRF ID14EH4 | ESRF ID14EH4 |
| Wavelength (Å) | 0.9795 | 0.9795 |
| Resolution range (Å) | 37.55-1.70 | 40.10-1.58 |
| (highest-resolution shell values) | (1.73-1.70) | (1.61-1.58) |
| Observations | 208685 (11143) | 240121 (11125) |
| Unique reflections | 48722 (2554) | 59724 (2925) |
| Completeness (%) | 99.8 (99.9) | 99.7 (99.7) |
| Multiplicity | 4.3 (4.4) | 4.0 (3.8) |
| R_merge_ (%) | 5.7 (51.3) | 5.8 (47.9) |
| I/σ(I) | 8.4 (1.3) | 7.0 (1.4) |
| Mean I/σ(I) | 13.5 (2.3) | 49.1 (0.8) |
| CC_1/2_^[a]^ | 0.998 (0.762) | 0.997 (0.724) |
| Average Mosaicity (º) | 0.57 | 0.37 |
|  |  |  |
| Refinement |  |  |
| No. of amino acids S6K-PKA | 338 | 337 |
| No. of amino acids PKI peptide | 20/20 | 19/20 |
| No. of water molecules | 556 | 633 |
| No. of Methanol molecules | - | 3 |
| No. of MPD molecules | 1 | - |
| No. of MEGA-8 molecules | - | - |
| No. of glycerol molecules | - | - |
| Ligand bound to active site | Compound **21a** | Compound **21e** |
| R-factor (%) | 15.5 | 15.3 |
| R_free_^a^ (%) | 17.9 | 17.8 |
|  |  |  |
| Ramachandran plot (%)^[b]^ |  |  |
| Favoured | 98 | 98 |
| Outliers | 0.3 | 0.3 |
|  |  |  |
| RMSD bonds | 0.01 | 0.01 |
| RMSD angles | 0.93 | 0.92 |

[a] P. A. Karplus, K. Diederichs, *Science* **2012**, *336*, 1030-1033.

[b] V. B. Chen, W. B. Arendall, 3rd, J. J. Headd, D. A. Keedy, R. M. Immormino, G. J. Kapral, L. W. Murray, J. S. Richardson, D. C. Richardson, *Acta Crystallogr. Sect. D* **2010**, *66*, 12-21.
